# Immune–related biomarkers shared by inflammatory bowel disease and liver cancer

**DOI:** 10.1371/journal.pone.0267358

**Published:** 2022-04-22

**Authors:** Thong Ba Nguyen, Duy Ngoc Do, Thuy T. P. Nguyen, Truc Ly Nguyen, Tung Nguyen-Thanh, Ha Thi Nguyen

**Affiliations:** 1 Department of Anatomy, Biochemistry, and Physiology, John A. Burns School of Medicine, University of Hawaii at Manoa, Honolulu, HI, United States of America; 2 Department of Animal Science and Aquaculture, Dalhousie University, Truro, Nova Scotia, Canada; 3 Department of Animal Science, Chonnam National University, Gwangju, Republic of Korea; 4 Department of Agricultural Biotechnology and Research Institute of Agriculture and Life Sciences, Seoul National University, Seoul, Republic of Korea; 5 Institute of Biomedicine, Hue University of Medicine and Pharmacy, Hue University, Hue, Vietnam; 6 Faculty of Basic Science, Hue University of Medicine and Pharmacy, Hue University, Hue, Vietnam; 7 Center for Molecular Biology, College of Medicine and Pharmacy, Duy Tan University, Danang, Vietnam; BMSCE: BMS College of Engineering, INDIA

## Abstract

It has been indicated that there is an association between inflammatory bowel disease (IBD) and hepatocellular carcinoma (HCC). However, the molecular mechanism underlying the risk of developing HCC among patients with IBD is not well understood. The current study aimed to identify shared genes and potential pathways and regulators between IBD and HCC using a system biology approach. By performing the different gene expression analyses, we identified 871 common differentially expressed genes (DEGs) between IBD and HCC. Of these, 112 genes overlapped with immune genes were subjected to subsequent bioinformatics analyses. The results revealed four hub genes (*CXCL2*, *MMP9*, *SPP1* and *SRC*) and several other key regulators including six transcription factors (*FOXC1*, *FOXL1*, *GATA2*, *YY1*, *ZNF354C* and *TP53*) and five microRNAs (miR-124-3p, miR-34a-5p, miR-1-3p, miR-7-5p and miR-99b-5p) for these disease networks. Protein-drug interaction analysis discovered the interaction of the hub genes with 46 SRC-related and 11 MMP9- related drugs that may have a therapeutic effect on IBD and HCC. In conclusion, this study sheds light on the potential connecting mechanisms of HCC and IBD.

## Introduction

Hepatocellular carcinoma (HCC), a major malignant form of the liver, is known as one of the most dangerous cancers and a leading cause of cancer deaths globally [1]. Surgical resection, transplantation and local ablation remain as standard therapeutic regimens for patients at an early stage of HCC with high overall survival (OS) rates. HCC patients with later stages, on the other hand, are usually subscribed for radio-/chemotherapies with significantly poorer outcomes [2]. These findings suggest an urgent demand for novel early diagnostic biomarkers and clinical treatment guidance for HCC patients. Generally, HCC develops in patients with cirrhosis and chronic liver inflammation, which were driven by a hepatitis virus infection, alcohol consumption, long-term smoking and non-alcoholic fatty liver-associated diseases [3–6]. Emerging evidence has demonstrated a contributing role of gut microbiomes in HCC development. Accordingly, microbial dysbiosis and leaky gut stimulate the release of microbiota-associated metabolites, remarkably contributing to hepatic inflammation, fibrosis, cell growth and anti-apoptosis signals [7, 8].

Inflammatory bowel disease (IBD) is a chronic inflammation of the gastrointestinal tract, which includes Crohn’s disease (CD) and ulcerative colitis (UC) [9]. Despite of being different in the clinical features, the pathogenesis of CD and UC involves the same risk factors, such as genetic susceptibility and alteration in gut microbiome and immune response [9]. Abnormal gut microbiota, for example, may contribute to intestinal inflammation and immune response dysregulation that eventually result in IBD [9–11]. Moreover, recent researches have demonstrated that severe IBD can lead to gastrointestinal cancers [12, 13] as well as various extra-intestinal manifestations including cardiovascular diseases, immune-mediated diseases [14–17] and malignancies such as cholangiocarcinoma, lymphoma, melanoma [18–23]. Remarkably, HCC risk among patients with IBD, especially among those with CD, have been repeatedly reported [21, 24–26]. These findings suggested a potential cross-talk in the pathophysiological pathways of IBD and HCC that needs to be further elucidated.

Despite numerous efforts on decoding the fundamental signaling molecules, the biomarkers and mutual underlying molecular pathways between HCC and IBD remained poorly understood. Therefore, in the present study, we have applied a systems biology approach to discover common potential biomarkers and the underlying mechanisms, thereby providing new insights into the pathology and clarifying the mutual immunity mechanisms of IBD and HCC.

## Materials and methods

### Data query

**Fig 1:**
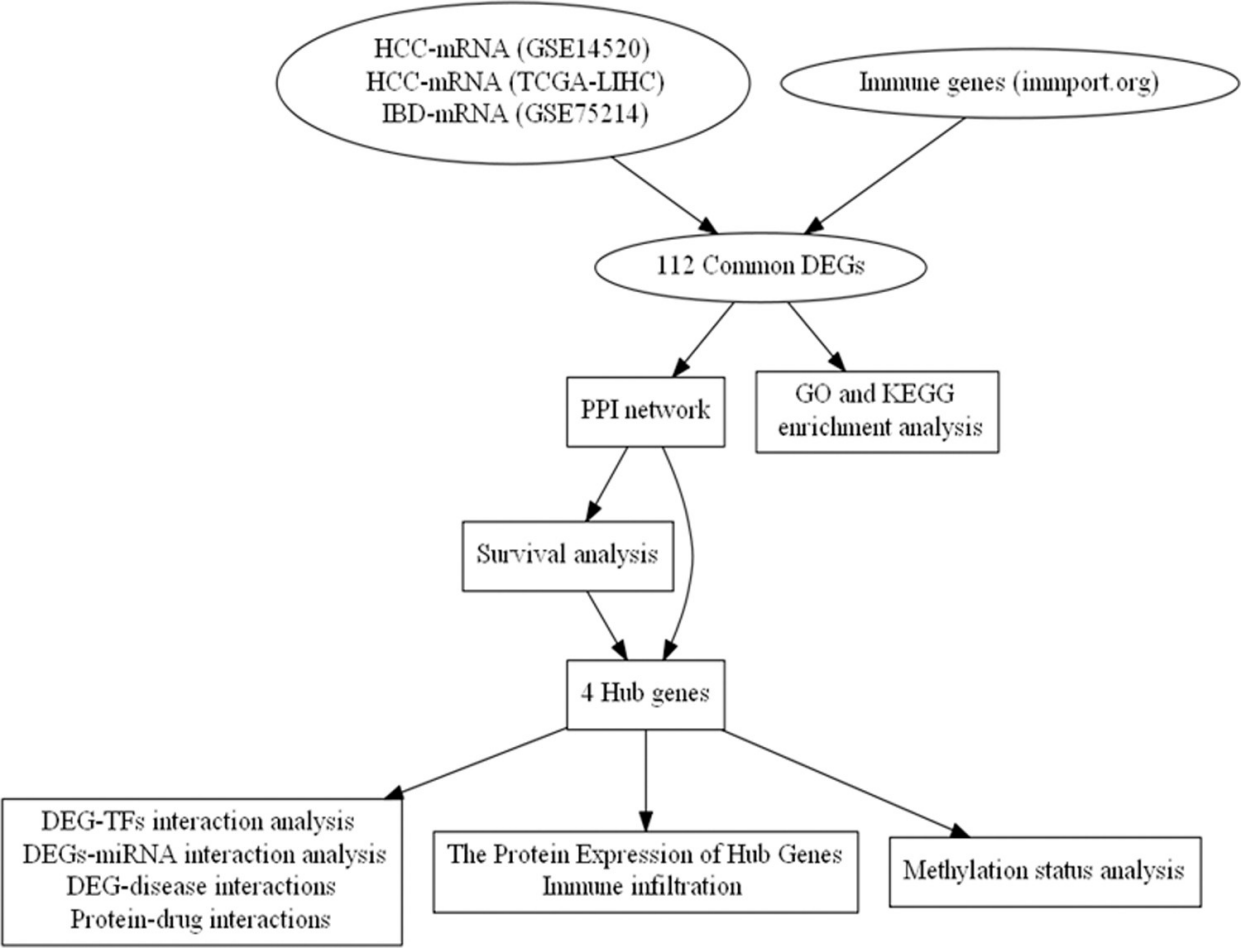
The workflow of the current study. HCC, hepatocellular carcinoma; IBD, inflammatory bowel disorder; DEGs, differentially expressed genes; PPI, protein-protein interaction; GO, gene ontology; KEGG, Kyoto Encyclopedia of Genes and Genomes; TFs, Transcription factors.

The workflow for the current study is presented in Fig 1. To discover the differentially expressed genes (DEGs) in IBD and HCC, three microarray datasets namely GSE75214 (https://www.ncbi.nlm.nih.gov/geo/query/acc.cgi?acc=GSE75214) for IBD and GSE14520 (https://www.ncbi.nlm.nih.gov/geo/query/acc.cgi?acc=GSE14520) and the Cancer Genome Atlas TCGA-LIHC (https://www.cancer.gov) for HCC were used as the input. IBD dataset (GSE75214) comprises the gene expression of biopsies from the colon of 11 controls, 97 UC and eight CD patients and from the (neo-)terminal ileum of 11 controls and 67 CD patients. The HCC dataset GSE14520 contains the gene expression of 225 HCC samples and 220 normal liver tissues and TCGA-LIHC includes 50 normal control and 374 HCC samples.

### Differential gene expression analysis

Prior to the differential gene expression analyses, data were transformed using log2 function in R program (ver. 4.0.2; R Development Core Team, Vienna, Austria). Then, a principal component analysis (PCA) was conducted using prcomp function to remove outliers. The genes (probes) that were expressed in less than three samples were excluded. The Limma [27] and DESeq2 packages [28] were used to find the significant DEGs and the *p*-values were corrected using the False Discovery Rate (FDR) correction toolkit in the R software (ver. 4.0.2; R Development Core Team, Vienna, Austria) for GSE and TCGA data. The significant DEGs with FDR < 0.05 for GSE (fold change > 1) and TCGA (fold change > 2) data were identified. A total of 1,793 immune genes (IMGs) were attained from 17 categories from the Analysis Portal (ImmPort) website (https://www.immport.org) and Immunology Database after excluding the duplicates [29]. The common DEGs between IBD, HCC and IMGs datasets were then identified and visualized by using VennDiagram package [30].

### Gene ontology and pathway enrichment analysis

Gene Ontology (GO) and Kyoto Encyclopedia of Genes and Genomes (KEGG) pathway via the clusterProfiler package [31] were used to assess the enrichment of common immune-related DEGs. A *p*-value < 0.01 was used as a cut-off to determine the significant enrichment GO terms and KEGG pathways.

### Identification of hub genes via protein-protein interaction (PPI) network construction

PPI network based on STRING (https://string-db.org/), an online tool for protein interaction analysis, was constructed for a set of common immune-related DEGs. *Homo sapiens* was selected as the organism for subsequent analysis. Network visualization in STRING was transferred to Cytoscape software (http://www.cytoscape.org/; version 3.8.2) to explore target modules and potential hub genes. The modules for potential hub genes in the PPI network via MCODE plugin were identified with MCODE score > 2 and nodes > 3 as the cut-off criteria. The interaction scores with a moderate confidence level of 0.4 was considered as the cut-off for constructive visualization and disjoint nodes were hidden. Degree > 21 was selected as a cut-off for hub gene identification.

### Survival analysis

The TCGA data of 347 patients with HCC were used for survival analysis. These HCC patients were assigned into low-risk and high-risk groups based on their median value of the prognostic risk score. Univariate Cox regression analysis was performed to explore the correlation between the DEGs and OS [32]. The hazard ratio (HR) of death was computed and Bonferroni adjusted *p*-values < 0.05 was considered statistically significant.

### The protein expressions of prognostic hub genes

The Human Protein Atlas (HPA; https://www.proteinatlas.org/) is an open-access resource for human transcriptome and proteome [33]. The hub genes were validated by immunohistochemistry results in HCC and normal tissues obtained from the HPA database and a previous study [34].

### The immune infiltration of prognostic hub genes

Since lymphocyte infiltration is an important indicator for lymph nodes’ status and cancer survival, the association between the hub genes expression levels and the immune infiltration levels in HCC was evaluated using TIMER version 1 (TIMER: Tumor Immune Estimation Resource) [35].

### Identification of DEGs-interacted transcription factors and microRNAs

To identify transcription factors (TFs) and microRNAs (miRNAs) that bind to the hub genes to regulate their expression, TF-target and miRNA-target interaction analyses were performed using two open-access databases, JASPAR [36] and MirTarbase [37], respectively, followed by a topological analysis using NetworkAnalyst [38].

### Protein-drug interaction analysis

Protein-drug interaction analysis provides information about the potential interaction between drugs and the target genes [39]. To identify potential drugs from the Comparative Toxicogenomics Database (CTD) that might interact with the common DEGs, protein-drug interaction analysis was performed via NetworkAnalyst [38].

### Gene-disease association analysis

The gene-disease associations by DisGeNET, which cover a wide range of biomedical characteristics of diseases, was commonly used to understand human genetic diseases [40]. The relationship between common DEGs and associated diseases was explored through NetworkAnalyst [38].

### DNA methylation analysis

The UALCAN tool was used to find the correlation between DNA methylation and four hub genes. It provided information on TCGA gene expression, DNA methylation, clinical data and friendly web resource [41].

## Results

### Common DEGs among HCC, IBD and IMGs

**Fig 2:**
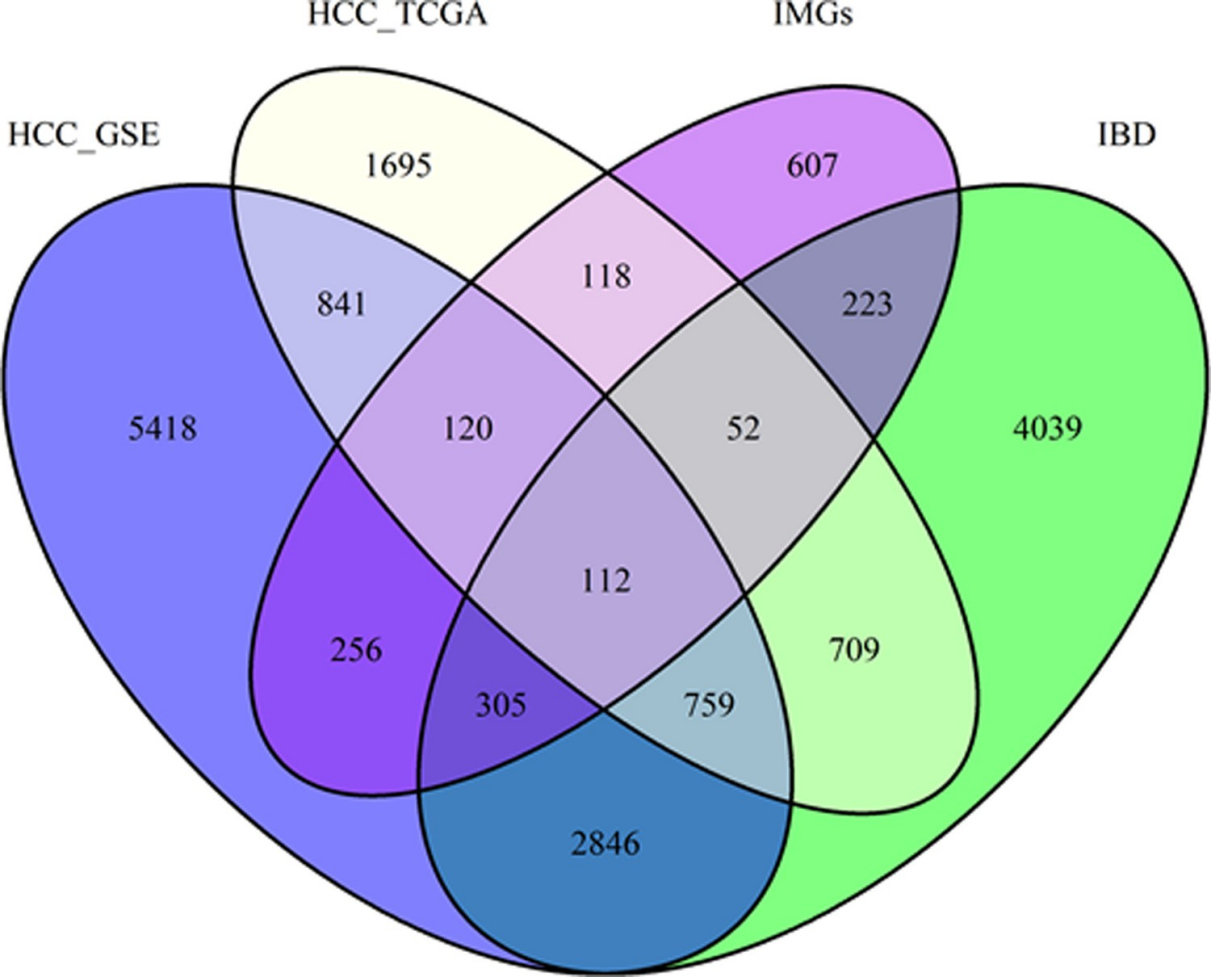
The Venn diagram for visualization of immune-related differentially expressed genes found in IBD, HCC. The value represented the number of unique gene symbols covered from the ensemble IDs and probe IDs. IBD, Inflammatory bowel disease; HCC, Hepatocellular carcinoma; IMGs, immune genes; TCGA, The Cancer Genome Atlas.

Gene expression datasets (HCC-GSE14520, HCC-TCGA and IBD-GSE75214) and IMGs list were collected in the current study. There were 9,045, 10,657, and 4,406 significant DEGs in the IBD-GSE75214, HCC-GSE14520, HCC-TCGA datasets, respectively. The Jvenn tool showed common DEGs among groups, 112 common DEGs among HCC, IBD and IMGs have been detected (Fig 2; S1 Table). These common DEGs were then subjected to further downstream analyses.

### Gene ontology and pathway enrichment analysis of common DEGs

**Fig 3:**
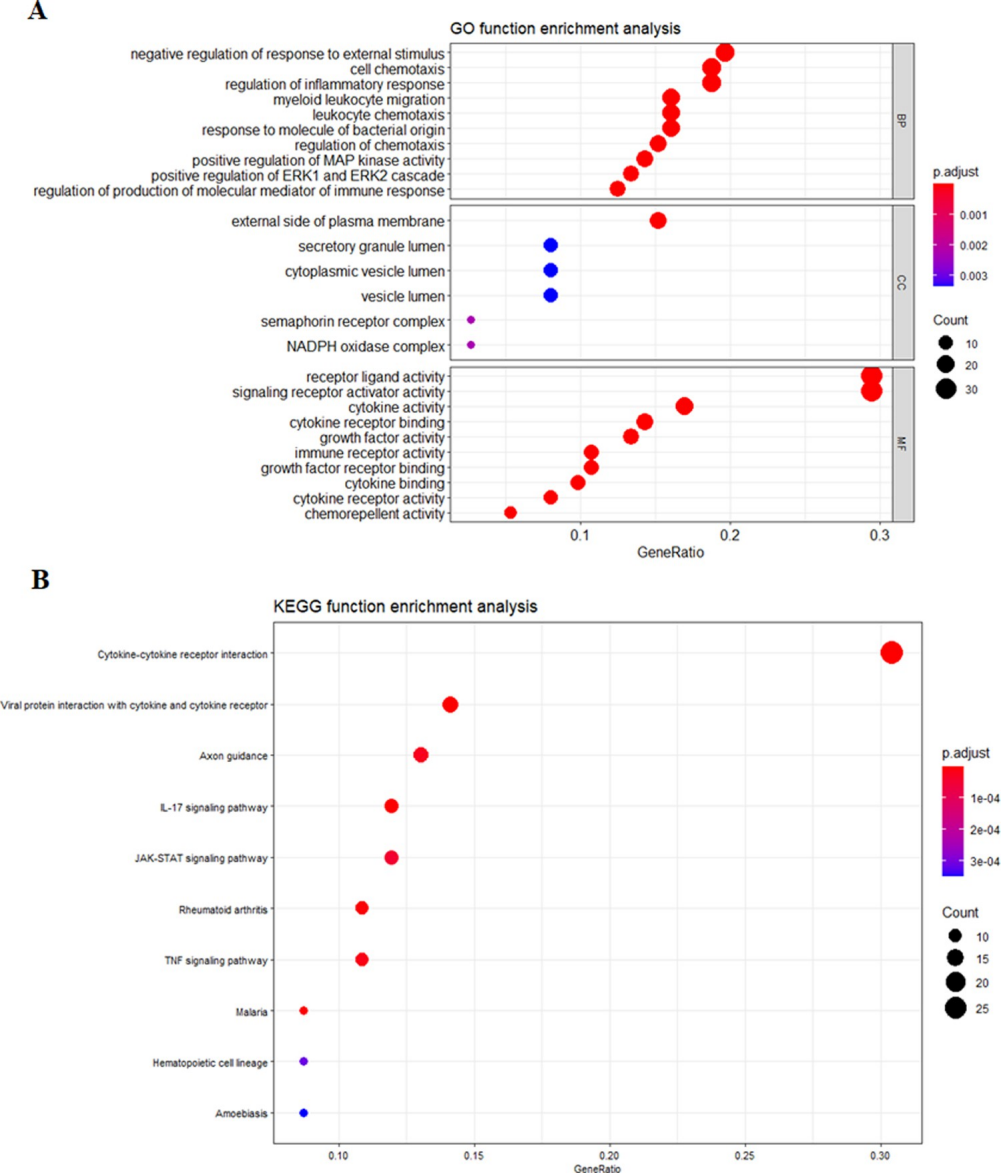
The mainly enriched (A) gene ontologies (GOs) and (B) KEGG pathways for 112 DEGs. The abscissa represents the number of genes enriched in the function. MF, molecular function; CC, cellular component; BP, biological process.

The top significantly enriched GOs and KEGG pathways were shown in Fig 3A and 3B, respectively. The GO analysis revealed that these common DEGs were significantly contributed to negative regulation of response to external stimulus, cell chemotaxis and regulation of inflammatory response under biological process (Fig 3A). For cellular component-GOs, common DEGs were significantly involved in the external side of the plasma membrane and secretory granule lumen. Lastly, for molecular function, DEGs were mainly involved in receptor-ligand activity, signaling receptor activator activity, cytokine activity, etc. (Fig 3A). In addition, the most importantly enriched KEGG pathways included cytokine-cytokine receptor interaction, viral protein interaction with cytokine and cytokine receptors, axon guidance, IL-17 signaling pathway in cancer (Fig 3B).

### Determination of hub proteins

**Fig 4:**
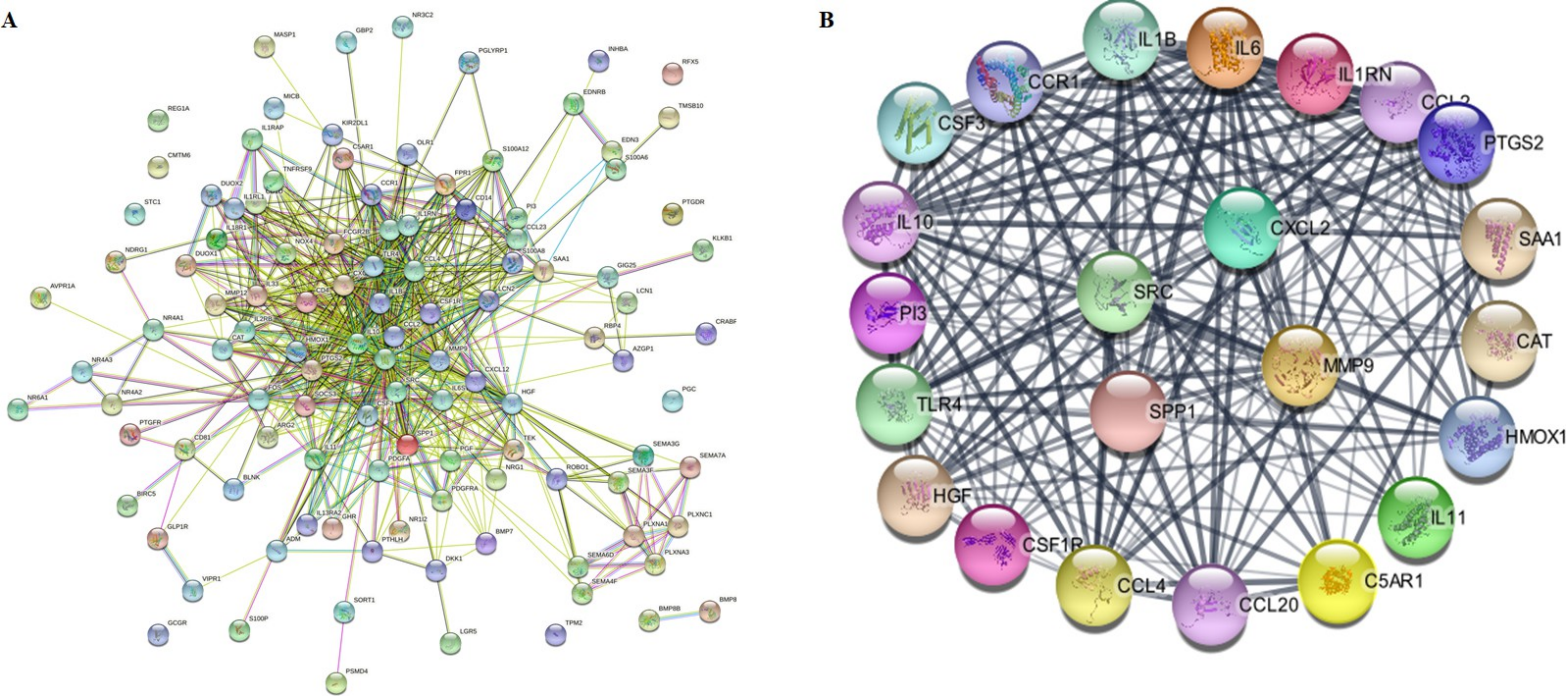
The protein-protein interaction (PPI) network of 112 immune-related differently expressed genes. The hub proteins were selected based on the topological parameter (degree>21). (A) The PPI network was generated using STRING. (B) The main module showed four hub genes in the center by MCODE plugin in the Cytoscape.

This PPI network analysis via STRING database is commonly used to investigate the biological responses in disease and health conditions. The PPI network of 112 common DEGs analysis revealed 20 hub genes that meet the cut-off degree > 21 (Fig 4A; S2 Table). Six modules for potential hub genes in the PPI network with MCODE score > 2 and nodes > 3 were identified (S1 Fig). The visualization of the main module using Cytoscape showed four hub proteins in the center, namely CXCL2, MMP9, SPP1 and SRC (Fig 4B). The topological features and involvement of the hub proteins in HCC and IBD were presented in Table 1.

**Table 1:** Overview of four hub proteins obtained from the protein-protein interaction network in HCC and IBD.

Symbol	Degree	Aspect
CXCL2	>21	• CXCL2 is downregulated in HCC. Overexpression of CXCL2 inhibits HCC cell proliferation and tumor growth; induces apoptosis [34].
		• CXCL2 was highly expressed in the inflamed colon of IBD patients [42, 43]. Overexpression of the corresponding receptor CXCR2 in mesenchymal stromal cells induces anti-inflammatory effect [42].
MMP9	>21	• MMP9 is associated with tumor invasion and poor outcomes and is expected to be a potential predictive marker for HCC patients [44].
		• MMP9 is upregulated in inflamed mucosa or serum of IBD patients and is a novel marker for intestinal inflammation [45].
SPP1	>21	• SPP1 promotes tumor growth in HCC; a diagnostic and therapeutic marker for HCC; SPP1 polymorphisms are associated with HCC occurrence [46, 47].
		• SPP1 is up-regulated in IBD. The SPP1 expression by CD103−dendritic cells (DCs) is crucial for their pathogenicity. Inhibiting the interaction of SPP1 with integrin α9 expressed on CD103−DCs abolished their inflammatory effects [48].
SRC	>21	• SRC promotes HCC progression, invasion and metastasis [49, 50].
		• c-SRC activity is highly induced in premalignant ulcerative colitis epithelia, and is strongly associated with colon cancer development [51].

HCC: Hepatocellular carcinoma; CXCL2: C-X-C Motif Chemokine Ligand 2; MMP9: Matrix Metalloproteinase 9; SPP1: Secreted Phosphoprotein 1; SRC: Proto-oncogene tyrosine-protein kinase Src.

### The hub genes validation

**Fig 5:**
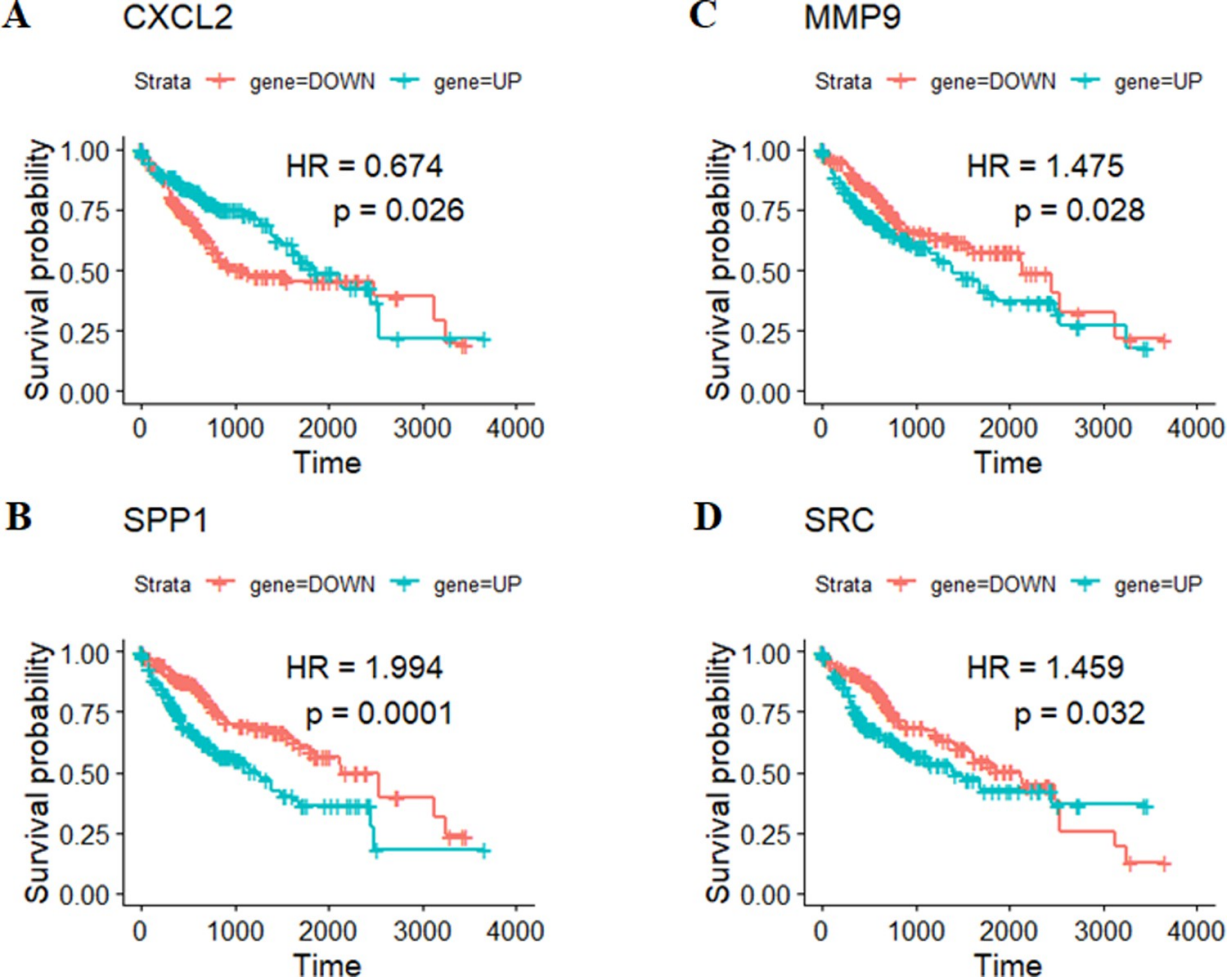
Association of the overall survival and four potential hub genes (A) *CXCL2*; (B) *MMP9*; (C) *SPP1*; (D) *SRC* in HCC based on Kaplan–Meier plotter. The horizontal axis signifies the time to event (in days). The patients were stratified into the high- and low-risk-level group and labeled with green and red color, respectively. HR is the hazard ratio of the high-risk over low-risk groups and *p* < 0.05 indicates a statistically significant difference.

The twenty hub genes from the PPI network with the highest degree were subjected to the survival analysis using univariate Cox analysis. The results exposed the significance of four hub genes (*CXCL2*, *MMP9*, *SPP1* and *SRC*) as prognostic makers of HCC (Fig 5; S3 Table). Specifically, the increased expression levels of *MMP9* (*p* = 0.028), *SPP1* (*p* = 0.0001) and *SRC* (*p* = 0.032) and the decreased expression levels of *CXCL2* (*p* = 0.026) were strongly related to poorer prognosis in HCC patients (Fig 5).

### The hub genes immunohistochemistry expression

**Fig 6:**
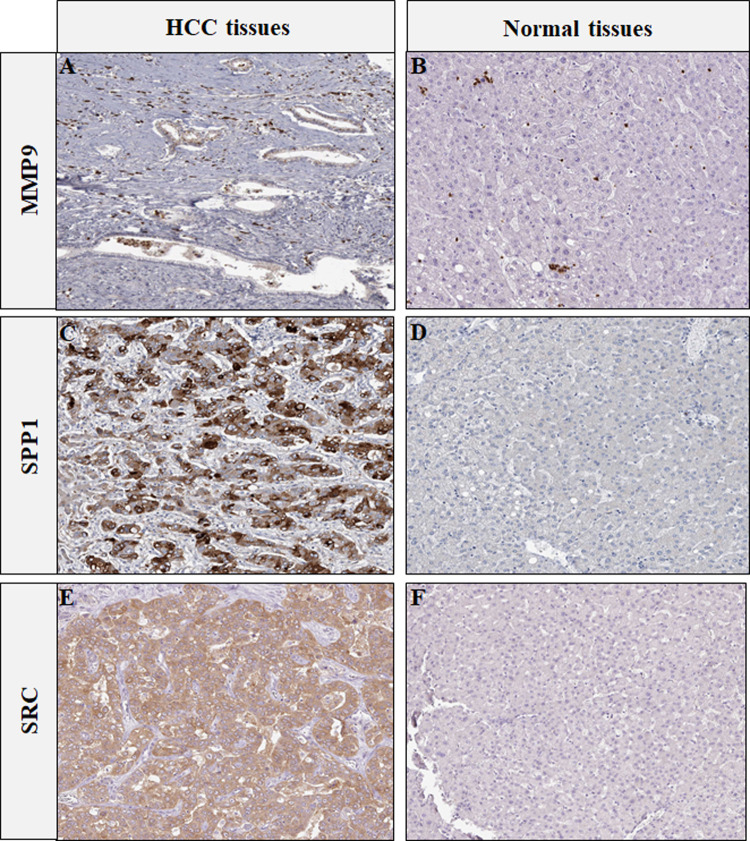
Immunohistochemistry of three hub proteins from the Human Protein Atlas database. Protein levels of (A) MMP9 in HCC tissues; (B) MMP9 in normal liver tissues; (C) SPP1 in HCC tissues; (D) SPP1 in normal liver tissues; (E) SRC in HCC tissues; (F) SRC in normal liver tissues.

The protein expression levels of four hub genes (*CXCL2*, *MMP9*, *SPP1* and *SRC*) in HCC and control group was explored via the HPA database and a previous study [34]. Accordingly, protein expression levels of MMP9, SPP1 and SRC were substantially increased and CXCL2 was decreased in HCC compared to the controls (Fig 6).

### The correlation between hub genes and immune cell infiltration and their methylation status in HCC

We used TIMER online tool to explore association between hub genes and six immune cell types (CD4+/CD8+ T cells, B cells, macrophages, neutrophils and dendritic cells) and tumor purity by the Spearman tests. These analyses indicated that MMP9 expression was significantly correlated infiltrating levels of all six immune cell types and tumor purity, especially high for dendritic cell, B cell, macrophage and CD8+ T cells; SPP1 expression was significantly associated with infiltrating levels of macrophage and dendritic cell; and SRC expression was significantly linked to macrophage, dendritic cell, CD4+ T cells, B cell and neutrophil (S2 Fig).

**Fig 7:**
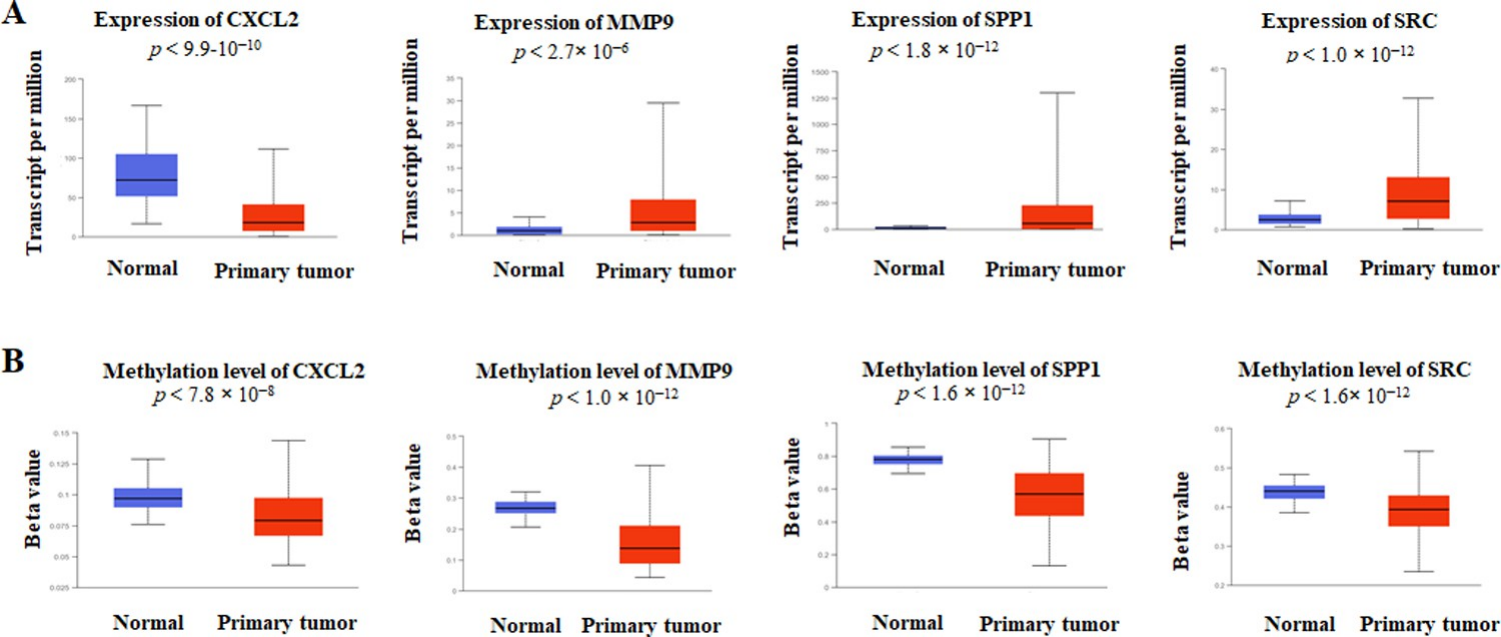
The expression and methylation status of hub genes in hepatocellular carcinoma (*n* = 377) as compared to the normal controls (*n* = 50) using TCGA samples. (A) Expression levels of *CXCL2*, *MMP9*, *SPP1* and *SRC* using TCGA samples; (B) Promoter methylation levels of *CXCL2*, *MMP9*, *SPP1* and *SRC*, respectively.

In addition, gene expression and methylation analysis showed significant differences in both gene expression (Fig 7A) and methylation patterns (Fig 7B) of *CXCL2*, *MMP9*, *SPP1* and *SRC* between liver tumor and normal liver tissue samples. Furthermore, a negative correlation between methylation patterns and gene expression was also noted for three genes (*MMP9*, *SPP1* and *SRC)*. This finding indicated that upregulation of these three hub genes might be a result of their diminished DNA methylation in HCC.

### Determination of regulatory signatures

**Fig 8:**
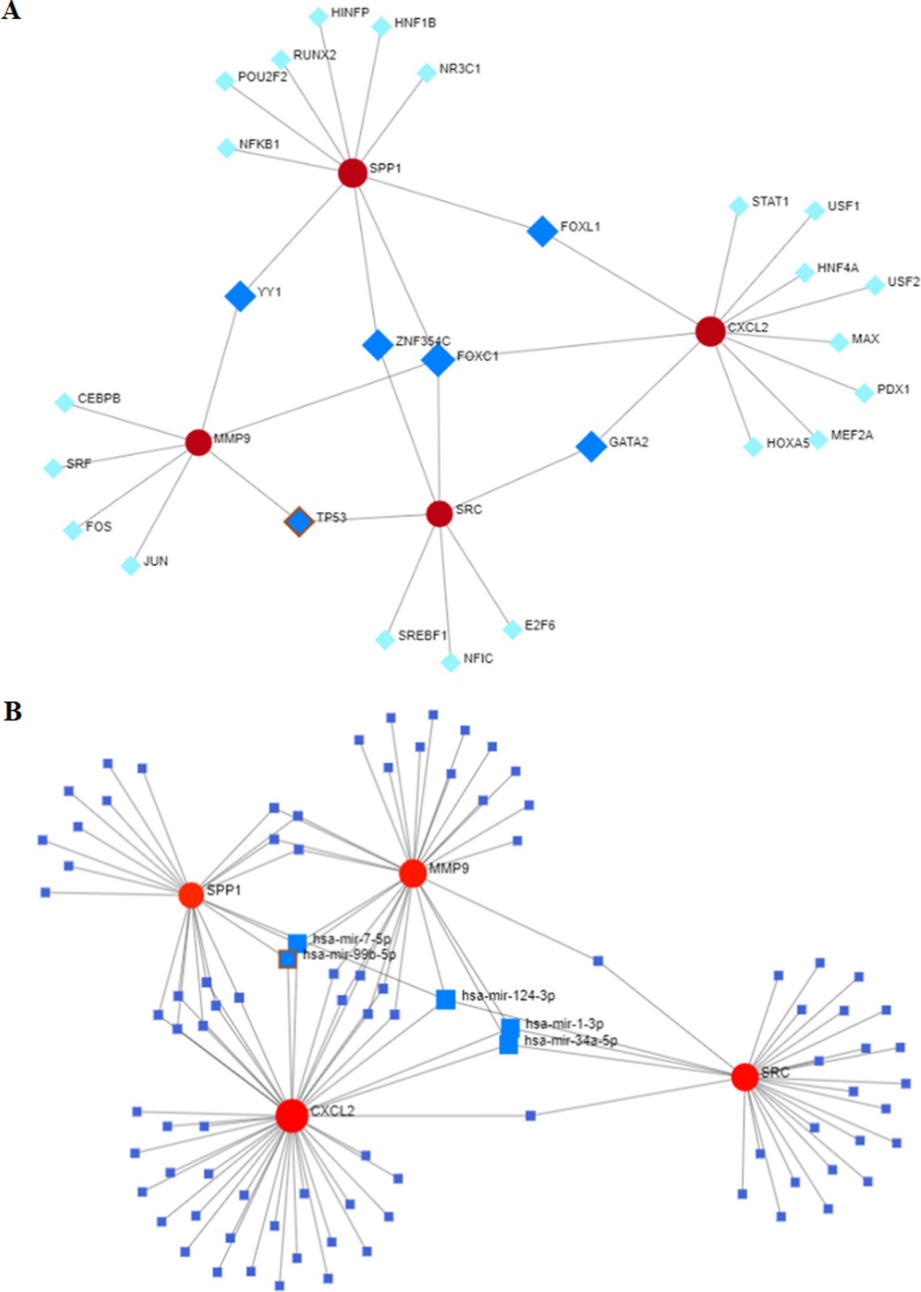
Hub gene interaction network. (A) Hub genes—transcription factors (TFs): The red spheres represent four hub genes; the blue squares represent six major hub genes-associated TFs and the cyan squares represent other gene-associated TFs; (B) Hub genes—miRNAs: red spheres represent four hub genes; the bigger blue squares show five main hub gene-associated miRNAs; the smaller blue squares show other hub gene-associated miRNAs.

Next, a network-based approach was performed to screen for the DEG-TF, DEG-miRNA interactions, thereby detecting the potential regulatory molecules of the hub DEGs. The gene-TF and gene-miRNA networks revealed six TFs namely *FOXC1*, *FOXL1*, *GATA2*, *YY1*, *ZNF354C* and *TP53* (Fig 8A; S4 Table) and five miRNAs namely miR-124-3p, miR-34a-5p, miR-1-3p, miR-7-5p and miR-99b-5p (Fig 8B; S5 Table) as the potential regulators of the four hub genes. The biological functions of these TFs and miRNAs in HCC are presented in Table 2.

**Table 2:** Top regulatory signatures of common DEGs predicted from DEG-TF and DEG-miRNA interaction networks.

Factors	Biological functions	References
**Transcription factors**		
*FOXC1*	Overexpression FOXC1 promotes HCC proliferation and metastasis	[52, 53]
*FOXL1*	Upregulation of FOXL1 inhibits proliferation and migration of HCC cells.	[54]
*GATA2*	Decreased expression of GATA2 was correlated with poor prognosis of HCC.	[55]
*YY1*	*YY1* is upregulated in HCC cell lines, which promotes tumor progression and inhibits cell differentiation in HCC. YY1 promotes the malignancy of HCC by increasing the expression of Quaking that is associated with poor outcomes of HCC.	[56–59]
*ZNF354C*	*ZNF354C* is a transcriptional repressor in HCC; patients with higher expression levels of ZNF354C exhibit a better overall survival.	[60]
*TP53*	*TP53* is a well-known tumor suppressor gene that was mutated in >30% of HCC patients. HCC patients with *TP53* hot-spot mutations (R249S and V157F) have poorer prognosis.	[61, 62]
**microRNAs**		
miR-124-3p	MiR-124-3p is a putative tumor suppressor whose expression was often reduced in HCC cells and tissues. It inhibits the proliferation, invasion and metastasis of HCC and is being considered as a novel diagnostic marker and therapeutic target for HCC	[63–65]
miR-34a-5p	MiR-34a-5p was usually downregulated in liver cancer cells and tissues. Its overexpression inhibits HCC cells growth and progression, while enhances apoptosis in HCC cells.	[66–68]
miR-1-3p	MiR-1-3p is downregulated in HCC cell lines. Overexpression of miR-1-3p induces apoptosis and inhibits cell proliferation in HCC. Serum miR-1 is a novel prognostic marker for patients with HCC.	[69, 70]
miR-7-5p	MiR-7-5p has been characterized as a tumor suppressor. It is generally downregulated in HCC tissues. Its overexpression suppresses tumor growth and metastasis. It can serve as a potential prognostic and/or therapeutic target for HCC.	[71–73]
miR-99b-5p	MiR-99b is highly expressed in HCC tissues and cell lines. Overexpression of miR-99b promotes tumor progression, migration and invasion of HCC and is associated with poor outcomes of patients with HCC.	[74]

HCC: Hepatocellular carcinoma; FOXC1: Forkhead Box C1; FOXL1: Forkhead Box L1; GATA2:Endothelial Transcription Factor GATA-2; YY1: Transcriptional Repressor Protein YY1; ZNF354C: Zinc Finger Protein 354C; TP53: Tumor Protein P53.

### Recognition of protein-drug and gene-disease interactions

To find drugs that can target the hub proteins, we studied protein-drug interactions and identified 46 SRC-related and 11 MMP9-related drugs (S3 Fig; S6 and S7 Tables). In addition, the gene-disease analysis showed that three out of four hub genes (*CXCL2*, *SRC* and *SPP1*) were also associated with mammary neoplasms, pulmonary fibrosis, dermatitis and allergic contact diseases (S4 Fig; S8 Table).

## Discussion

Despite the immune system has been well recognized for its roles in regulating tumorigenesis, there are no effective molecular targets currently available in routine clinical practice since the exact mechanisms involved in its pathogenesis remain poorly understood [75–77]. The immunotherapy remains unclear in liver cancer; therefore, it is necessary to classify the potentially effective patients who might be benefit from the therapy and to predict the outcomes. IBD itself alters the gut microbiome [78, 79]. The harmful bacteria were then directed to portal circulation, causing abnormal expression of cell adhesion molecules, thereby increasing the risk of liver cancer [25]. Furthermore, the treatment therapies of IBD might also stimulate the HCC progression related to the impairment of the immune response [12]. In this study, we identified 112 common DEGs among IBD, HCC datasets and an immune gene list. Both GO and KEGG pathway analyses revealed a significant role of the inflammatory response in the HCC progression, in which cytokine-cytokine receptor interaction is the most common between IBD- and HCC-associated genes. In addition, this interaction was reported to be remarkably associated with HCC, supporting our observation [80, 81].

The current study identified *CXCL2*, *MMP9*, *SPP1* and *SRC* as the four hub genes among IBD, HCC and IMGs. Notably, *CXCL2*, a small cytokine of the CXC chemokine family, was identified as a top significant gene through PPI network, IHC staining and survival analysis. CXCL2 related to neutrophil response under various conditions such as wound healing, cancer metastasis and angiogenesis [82]. Recently, *CXCL2* was reported as an inhibitor of the HCC cell cycle [34]. Exosomes containing CXCL2 or expressing CXCL2 receptors improved chemotaxis of HCC; thus, it was exploited for targeted drug delivery. Additionally, CXCL2 was also noted to be an important cytokine for IBD [43]. Particularly, CXCR2, mediated the release of neutrophils from the bone marrow via binding to its two ligands (CXCL1, CXCL2) [83].

*MMP9*, *SPP1* and *SRC*, in contrast, were positive regulators of HCC cell death. In this study, *SPP1* is the most significant interaction gene of HCC and IBD. Osteopontin, a protein encoded by the *SPP1* gene, is up-regulated in IBD [84]. SPP1, with inference value of 129.66 from the gene-disease association dataset, is regarded as potential drug targets for the liver cancer treatment. Moreover, SPP1 promotes HCC growth and induces resistance to cell apoptosis, suggesting that SSP1 is a potential therapeutic target in HCC [46]. MMP9 plays a vital role in promoting cell migration and metastasis [85]. HCC develops as a result of a change in MMP 9 protein expression [86]. Lastly, *SRC*, which belongs to a group of SRC family kinases, primarily involved in the regulation of embryonic development and cell growth. A previous study showed that increased SRC expression and activity promoted cancer progression processes, including cell proliferation, differentiation, invasion and migration [87]. In HCC, SRC signaling pathway contributes to cell growth, metastasis and drug resistance via targeting ASPP2, TIGF, L-FABP, GRP78, CD47 and TM4SF5/CD44 [87]. Saracatinib, an SRC inhibitor, might improve the outcomes of liver cancer patients and have been approved by the food and drug administration organization for the treatment of HCC [88]. Moreover, the IHC staining results showed an increase of MMP9, SPP1 and SRC protein expression levels and a significant decrease in CXCL12 [34] in HCC tissues. Besides, our results showed a moderate positive association between four hub genes and infiltration levels of macrophages and dendritic cells. These outcomes were supported by GO results, suggesting the possible regulatory role of the target genes in negative response with an external stimulus of tumor-related to those cells. Likewise, these results indicated that three hub genes can activate macrophages, resulting in increased T cell exhaustion. Moreover, this study discovered negative correlations between the gene expression levels and methylation status of three hub genes (*MMP9*, *SPP1* and *SRC*). Interestingly, when the expression levels of three hub genes significantly increased, their methylation profile was significantly inhibited. In addition, the upregulation of SPP1 and SRC due to lower promoter methylation in liver carcinoma has been reported [89, 90], which is in agreement with the findings of this study.

Different interaction networks have been analyzed to identify the potential regulatory miRNAs and TFs of the hub genes. Among the identified TFs, FOXC1 was considered as a novel biomarker for the early stages of HCC [53]. Foxl1 was able to induce liver repair by activating the canonical Wnt/b-catenin pathway [91]. Reduced GATA2 expression was related to a poor outcome of HCC following resection [55]. YY1 enhanced linc01134 transcription by interacts with linc01134 promoter to mediates HCC progression [92]. In addition, YY1 has been shown to be an important mediator of the mTORC1, a signaling pathway in immune cell metabolism [93]. ZNF354C play a vital role in the dissociation of the complex from CHD1L and BCL9 promoters to abolish the transcription inhibition, suggesting its potential target for diagnosis and treatment of HCC [60]. *TP53*, a tumor suppressor gene, plays a critical function for the HCC progression [94].

MiRNAs are short (~22nt) RNA molecules that directly bind to the target mRNAs and negatively regulate their expression. MiRNAs are extensively studied and being used as potential biomarkers for various human diseases, including cancers. This study identified five potential miRNAs (miR-124-3p, miR-1-3p, miR-7-5p, miR-34a-5p and miR-99b-5p) that might target and regulate the expression of four hub genes. These five miRNAs are downregulated in HCC (Table 2) [66, 69, 71, 95]. Additionally, these five miRNAs were also suggested as the molecular signature of IBD [96–101]. For instance, miR-7-5p inhibited the expression of *TFF3* in IBD [101].

Protein-drug interaction analysis is central to drug discovery and disease treatment, which contributes to understanding the mechanisms of action and potential side effects of drugs, as well as the sensitivity of the receptors [102]. The current study identified 46 SRC-related and 11 MMP9-related drugs. Additionally, the protein-disease interaction networks showed three out of five hub genes (*CXCL2*, *SRC* and *SPP1*) are also associated with mammary neoplasms, pulmonary fibrosis, dermatitis and allergic contact diseases. The presence of the common genes between conditions suggested that there might be a link between them [103]. However, in the present study, the identification of the hub genes was barely based on the gene expression microarray data overlapping with immune genes, which might cause some errors/biases in the outcomes. Additional experimental evidence is required to confirm the findings.

## Conclusions

In summary, these results strongly indicated *CXCL2*, *MMP9*, *SPP1* and *SRC* as key genes in IBD and HCC. The analyses of the present study identified several TFs (*FOXC1*, *FOXL1*, *GATA2*, *YY1*, *ZNF354C* and *TP53*) and miRNAs (miR-124-3p, miR-1-3p, miR-7-5p, miR-34a-5p and miR-99b-5p) that potentially regulate those key genes. These hub genes and their transcriptional and/or posttranscriptional products might be the potential therapeutic targets in connecting mechanisms of HCC and IBD.

## Supporting information

S1 Fig Six modules (A-F) for potential hub genes in the protein-protein interaction networks by Cytoscape. (DOCX)

S2 Fig Correlation of hub genes expression with tumor purity and immune infiltration level in hepatocellular carcinoma. (A*) CXCL2*; (B) *MMP9*; (C) *SPP1*; (D) *SRC*.
(DOCX)

S3 Fig Protein-drug interactions by NetworkAnalyst. The red spheres show two hub proteins, (A) MMP9 and (B) SRC; the blue squares show the hub gene-associated drugs.
(DOCX)

S4 Fig Gene-disease interactions by NetworkAnalyst. The red spheres represent three hub genes; the bigger blue squares represent three main diseases associated with three hub genes and the smaller blue squares represent other diseases associated with three hub genes.
(DOCX)

S1 Table Differential gene expression analysis of 112 common DEGs in the TCGA-LIHC dataset. (DOCX)

S2 Table Protein-protein interaction network. (DOCX)

S3 Table The association of the top 20 hub genes and overall survival in the TCGA_LIHC dataset. (DOCX)

S4 Table Gene-transcription factor interaction network. (DOCX)

S5 Table Gene-microRNA interaction network. (DOCX)

S6 Table Gene-drug interaction network of SRC. (DOCX)

S7 Table Gene-drug interaction network of MMP9. (DOCX)

S8 Table Gene-disease interaction network. (DOCX)
