# Supplementary material for: Immune–related biomarkers shared by inflammatory bowel disease and liver cancer
Source: PLoS One. 2022 Apr 22;17(4):e0267358. doi: 10.1371/journal.pone.0267358 (PMC9032416; doi:10.1371/journal.pone.0267358)
Supplement: S1 Fig — (DOCX) [file pone.0267358.s001.docx]

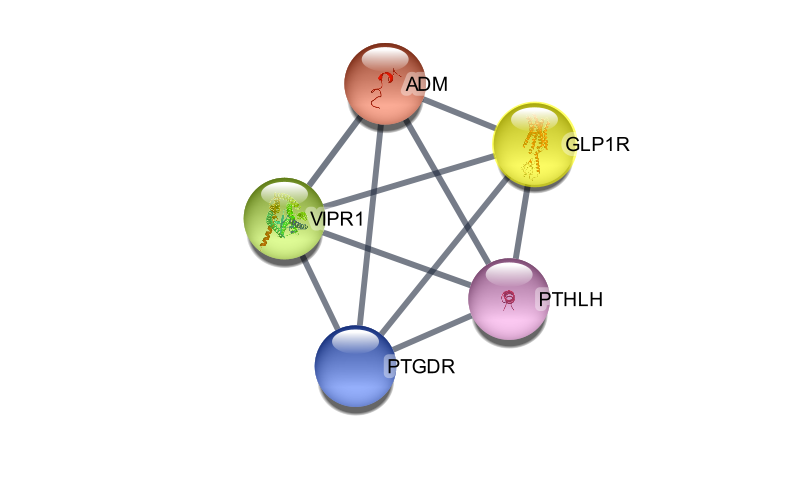

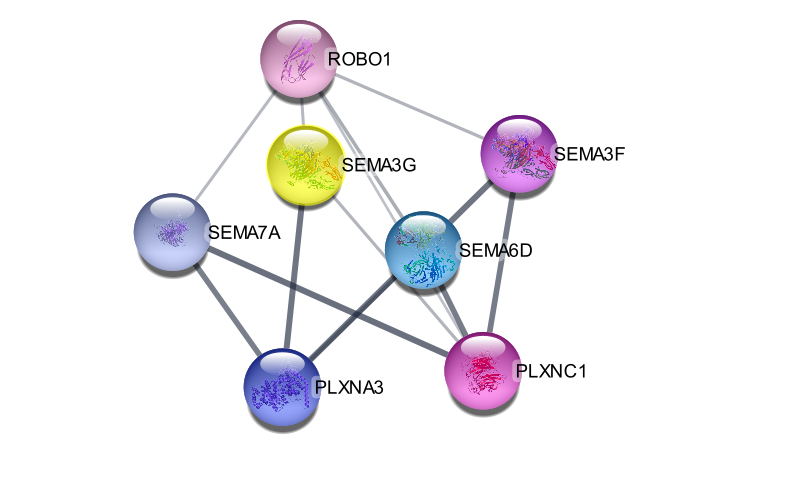

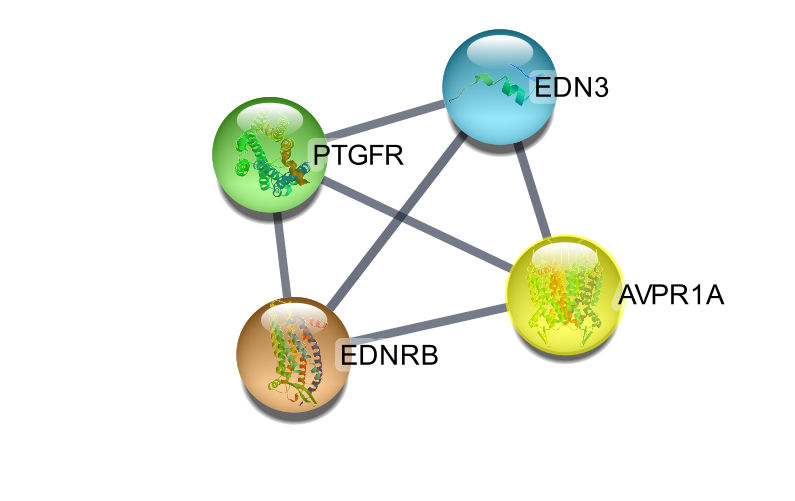

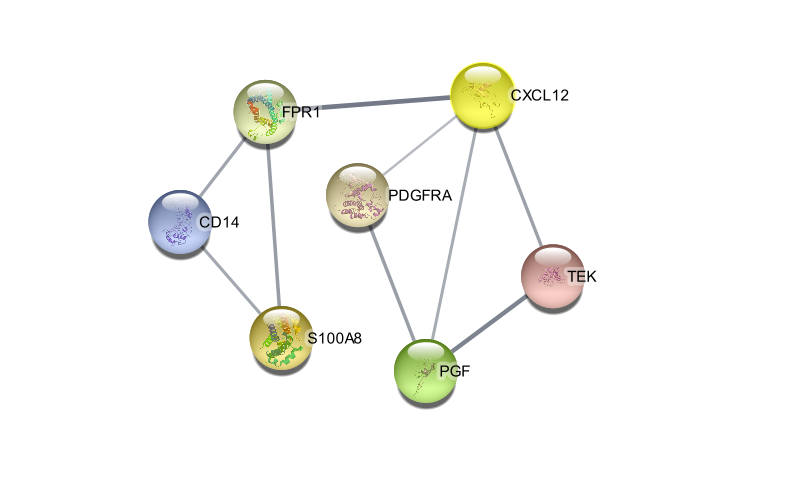

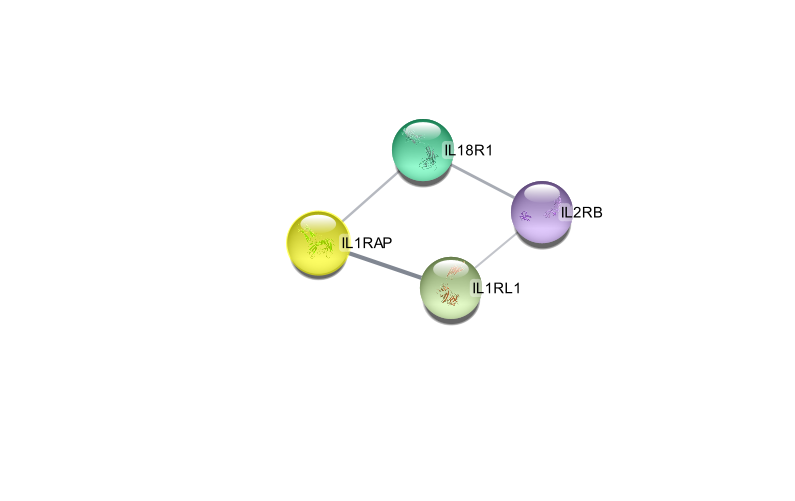

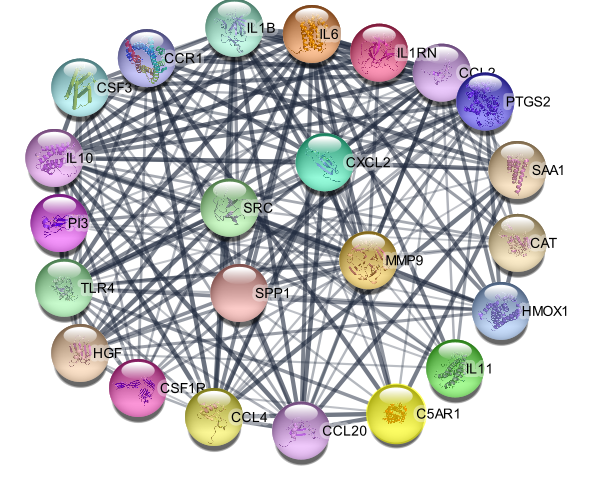


**D**

**A**

**B**

**C**

**E**

**F**

**S1 Fig. Six modules (A-F) for potential hub genes in the protein-protein interaction networks by Cytoscape.**
