# Supplementary material for: Immune–related biomarkers shared by inflammatory bowel disease and liver cancer
Source: PLoS One. 2022 Apr 22;17(4):e0267358. doi: 10.1371/journal.pone.0267358 (PMC9032416; doi:10.1371/journal.pone.0267358)
Supplement: S2 Fig — (A) CXCL2; (B) MMP9; (C) SPP1; (D) SRC. (DOCX) [file pone.0267358.s002.docx]

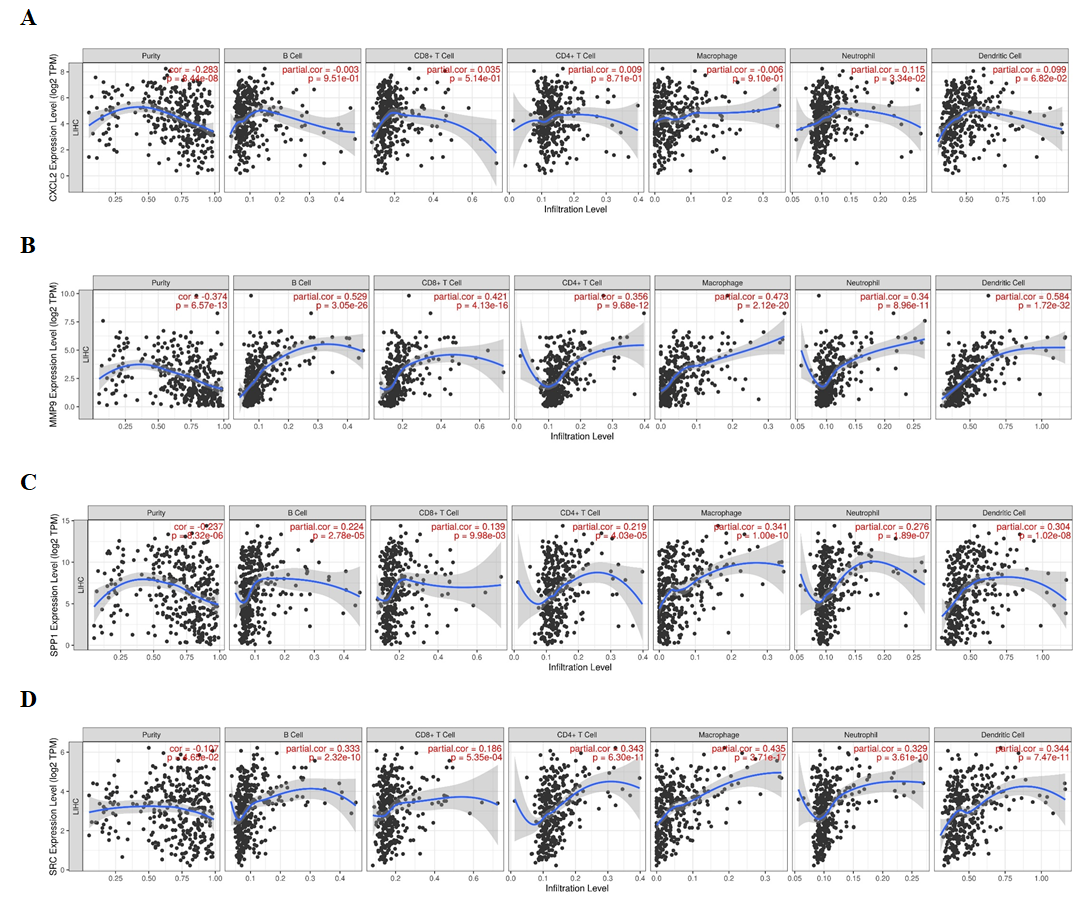


**S2 Fig.** **Correlation of hub genes expression with tumor purity and immune infiltration level in hepatocellular carcinoma**. (A*) CXCL2*; (B) *MMP9*; (C) *SPP1*; (D) *SRC*.
