# Supplementary material for: Immune–related biomarkers shared by inflammatory bowel disease and liver cancer
Source: PLoS One. 2022 Apr 22;17(4):e0267358. doi: 10.1371/journal.pone.0267358 (PMC9032416; doi:10.1371/journal.pone.0267358)
Supplement: S3 Fig — The red spheres show two hub proteins, (A) MMP9 and (B) SRC; the blue squares show the hub gene-associated drugs. (DOCX) [file pone.0267358.s003.docx]

**S3 Fig. Protein-drug interactions by NetworkAnalyst.** The red spheres show two hub proteins, (A) MMP9 and (B) SRC; the blue squares show the hub gene-associated drugs.


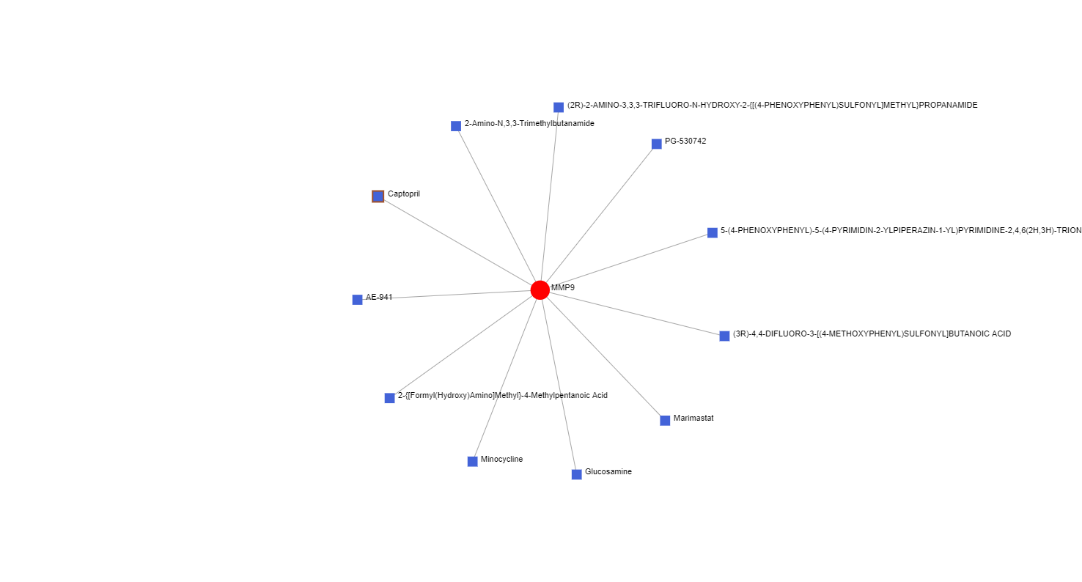

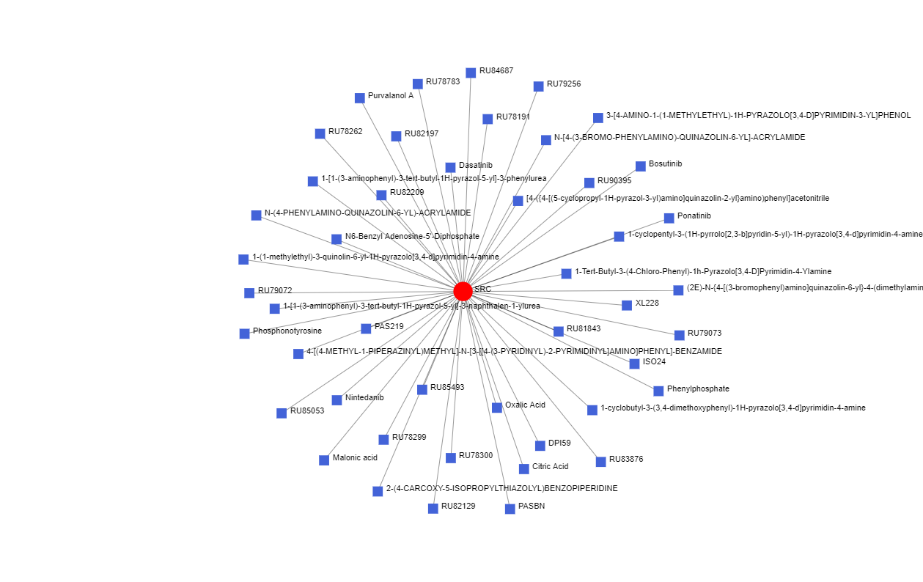


**A**

**B**
