# Supplementary material for: Immune–related biomarkers shared by inflammatory bowel disease and liver cancer
Source: PLoS One. 2022 Apr 22;17(4):e0267358. doi: 10.1371/journal.pone.0267358 (PMC9032416; doi:10.1371/journal.pone.0267358)
Supplement: S4 Fig — The red spheres represent three hub genes; the bigger blue squares represent three main diseases associated with three hub genes and the smaller blue squares represent other diseases associated with three hub genes. (DOCX) [file pone.0267358.s004.docx]

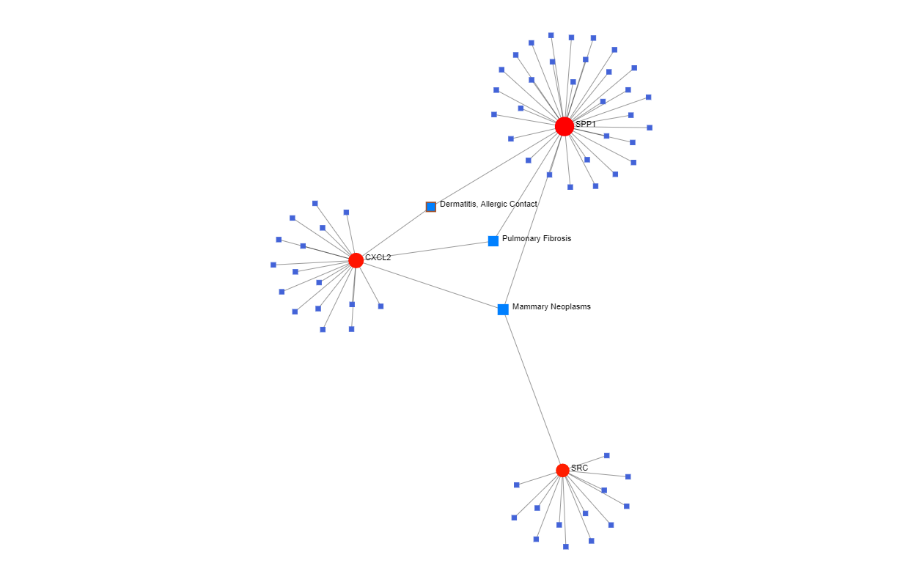


**S4 Fig. Gene-disease interactions by NetworkAnalyst.** The red spheres represent three hub genes; the bigger blue squares represent three main diseases associated with three hub genes, and the smaller blue squares represent other diseases associated with three hub genes.
