# Supplementary material for: Immune–related biomarkers shared by inflammatory bowel disease and liver cancer
Source: PLoS One. 2022 Apr 22;17(4):e0267358. doi: 10.1371/journal.pone.0267358 (PMC9032416; doi:10.1371/journal.pone.0267358)
Supplement: S1 Table — (DOCX) [file pone.0267358.s005.docx]

**S1 Table. Differential gene expression analysis of 112 common DEGs in the TCGA-LIHC dataset.**

| Gene | baseMean | log2FC | lfcSE | stat | *p* value | adj. *p* |
| --- | --- | --- | --- | --- | --- | --- |
| ADM | 1002.408217 | -1.344531485 | 0.178251197 | -7.5429 | 4.60E-14 | 3.41E-13 |
| ARG2 | 186.3420921 | 1.254281667 | 0.262717019 | 4.774269 | 1.80E-06 | 5.42E-06 |
| AVPR1A | 1641.201597 | -2.457945236 | 0.373960884 | -6.57273 | 4.94E-11 | 2.60E-10 |
| AZGP1 | 47406.61283 | -1.555143464 | 0.213630115 | -7.27961 | 3.35E-13 | 2.25E-12 |
| BIRC5 | 1038.39194 | 4.125388674 | 0.207771843 | 19.85538 | 9.90E-88 | 1.59E-84 |
| BLNK | 1068.77605 | -1.031224846 | 0.130029564 | -7.9307 | 2.18E-15 | 1.85E-14 |
| BMP7 | 25.11906475 | 3.915371849 | 0.545835621 | 7.17317 | 7.33E-13 | 4.74E-12 |
| BMP8A | 9.181603665 | 1.033939244 | 0.225678079 | 4.581478 | 4.62E-06 | 1.32E-05 |
| BMP8B | 137.5767643 | 1.441599932 | 0.271260127 | 5.314456 | 1.07E-07 | 3.78E-07 |
| C5AR1 | 404.2863269 | -1.107702152 | 0.156244444 | -7.08955 | 1.35E-12 | 8.44E-12 |
| CAT | 18905.00764 | -1.440491967 | 0.141089702 | -10.2098 | 1.79E-24 | 3.50E-23 |
| CCL2 | 767.95717 | -1.397106797 | 0.230512157 | -6.06088 | 1.35E-09 | 5.99E-09 |
| CCL20 | 1660.827746 | 1.314887638 | 0.317138166 | 4.146103 | 3.38E-05 | 8.63E-05 |
| CCL23 | 24.22515901 | -3.026235548 | 0.174699551 | -17.3225 | 3.18E-67 | 8.39E-65 |
| CCL4 | 256.5307795 | -1.315399984 | 0.204071982 | -6.44576 | 1.15E-10 | 5.80E-10 |
| CCR1 | 252.1780655 | -1.48698195 | 0.181407053 | -8.19694 | 2.47E-16 | 2.32E-15 |
| CD14 | 20300.68737 | -1.421490222 | 0.187797798 | -7.56926 | 3.75E-14 | 2.81E-13 |
| CD1D | 333.3301843 | -1.617230503 | 0.211719717 | -7.63854 | 2.20E-14 | 1.68E-13 |
| CD4 | 2801.149596 | -1.972919779 | 0.160522571 | -12.2906 | 1.02E-34 | 4.75E-33 |
| CD81 | 25555.83616 | -1.027445223 | 0.134287774 | -7.65107 | 1.99E-14 | 1.53E-13 |
| CMTM6 | 4053.889303 | -1.016948796 | 0.102221571 | -9.94848 | 2.56E-23 | 4.59E-22 |
| CRABP1 | 1.967103847 | 3.042231935 | 0.733053347 | 4.150083 | 3.32E-05 | 8.49E-05 |
| CSF1R | 1368.668442 | -1.033594676 | 0.173354529 | -5.96232 | 2.49E-09 | 1.07E-08 |
| CSF3 | 2.384939247 | -2.961158099 | 0.39286914 | -7.53726 | 4.80E-14 | 3.55E-13 |
| CXCL12 | 3323.141759 | -2.684606762 | 0.224629229 | -11.9513 | 6.39E-33 | 2.67E-31 |
| CXCL2 | 2096.695167 | -1.516059676 | 0.234022662 | -6.47826 | 9.28E-11 | 4.73E-10 |
| DKK1 | 767.3168672 | 5.935828423 | 0.465152181 | 12.76105 | 2.71E-37 | 1.47E-35 |
| DUOX1 | 162.554721 | 2.345448637 | 0.235547736 | 9.957424 | 2.34E-23 | 4.21E-22 |
| DUOX2 | 533.360497 | 3.31121016 | 0.44314856 | 7.472009 | 7.90E-14 | 5.72E-13 |
| EDN3 | 3.873643775 | 1.670006276 | 0.583735413 | 2.860896 | 0.004224 | 0.007837 |
| EDNRB | 1130.294989 | -1.467987021 | 0.153723806 | -9.54951 | 1.30E-21 | 2.02E-20 |
| FCGR2B | 335.3742609 | -1.664429042 | 0.295348263 | -5.63548 | 1.75E-08 | 6.79E-08 |
| FOS | 6102.560207 | -3.0903777 | 0.242950134 | -12.7202 | 4.57E-37 | 2.47E-35 |
| FPR1 | 135.6540362 | -1.966379572 | 0.233510571 | -8.42094 | 3.73E-17 | 3.80E-16 |
| GBP2 | 3917.901808 | 1.392570425 | 0.159282435 | 8.742775 | 2.27E-18 | 2.58E-17 |
| GCGR | 2091.520517 | -1.444713386 | 0.352257865 | -4.10129 | 4.11E-05 | 0.000104 |
| GHR | 3634.40696 | -2.515717619 | 0.21650993 | -11.6194 | 3.28E-31 | 1.18E-29 |
| GLP1R | 18.29923158 | 5.919296155 | 0.638851098 | 9.265533 | 1.94E-20 | 2.67E-19 |
| HGF | 577.2592403 | -2.022773297 | 0.278703457 | -7.2578 | 3.93E-13 | 2.63E-12 |
| HMOX1 | 3659.469085 | -1.352851224 | 0.20081308 | -6.73687 | 1.62E-11 | 9.03E-11 |
| IL10 | 14.22485544 | -1.551154303 | 0.229565651 | -6.75691 | 1.41E-11 | 7.93E-11 |
| IL11 | 6.428750314 | 1.088376408 | 0.302351875 | 3.599701 | 0.000319 | 0.000707 |
| IL13RA2 | 97.82974827 | -1.600107354 | 0.366398789 | -4.36712 | 1.26E-05 | 3.40E-05 |
| IL18R1 | 225.8681536 | -1.483264393 | 0.183012318 | -8.10472 | 5.29E-16 | 4.80E-15 |
| IL1B | 70.14359406 | -1.781774508 | 0.230827985 | -7.71906 | 1.17E-14 | 9.21E-14 |
| IL1RAP | 1904.482563 | -2.060249466 | 0.16821451 | -12.2478 | 1.73E-34 | 7.84E-33 |
| IL1RL1 | 78.81477309 | -3.540024766 | 0.307099618 | -11.5273 | 9.61E-31 | 3.35E-29 |
| IL1RN | 3334.805538 | -2.126415583 | 0.199183188 | -10.6757 | 1.32E-26 | 3.13E-25 |
| IL2RB | 380.0194521 | -1.119092501 | 0.199548319 | -5.60813 | 2.05E-08 | 7.88E-08 |
| IL33 | 531.8704324 | -1.5783956 | 0.219907347 | -7.17755 | 7.10E-13 | 4.61E-12 |
| IL6 | 40.28678128 | -1.951034688 | 0.320897035 | -6.07994 | 1.20E-09 | 5.35E-09 |
| IL6ST | 10134.55258 | -1.008540313 | 0.097386436 | -10.3561 | 3.93E-25 | 8.12E-24 |
| INHBA | 629.3610303 | -1.016994994 | 0.214131256 | -4.7494 | 2.04E-06 | 6.09E-06 |
| KIR2DL1 | 2.244516817 | -1.572168685 | 0.288227315 | -5.45461 | 4.91E-08 | 1.81E-07 |
| KLKB1 | 5107.240517 | -1.625722164 | 0.18810677 | -8.64255 | 5.50E-18 | 6.01E-17 |
| LCN1 | 0.679755017 | 1.621111139 | 0.546925696 | 2.964043 | 0.003036 | 0.005782 |
| LCN2 | 10059.04637 | 4.769023912 | 0.37641486 | 12.6696 | 8.72E-37 | 4.59E-35 |
| LGR5 | 510.8971909 | 2.150669016 | 0.424172935 | 5.070265 | 3.97E-07 | 1.30E-06 |
| MASP1 | 6084.206478 | -1.632759221 | 0.163021758 | -10.0156 | 1.30E-23 | 2.39E-22 |
| MICB | 197.5252373 | 1.299435246 | 0.166067746 | 7.82473 | 5.09E-15 | 4.16E-14 |
| MMP12 | 74.73811917 | 4.140823402 | 0.419741755 | 9.865169 | 5.89E-23 | 1.02E-21 |
| MMP9 | 824.7105961 | 2.13343697 | 0.293587992 | 7.266772 | 3.68E-13 | 2.47E-12 |
| NDRG1 | 10467.62918 | 1.225222909 | 0.19376144 | 6.323358 | 2.56E-10 | 1.24E-09 |
| NOX4 | 59.50118567 | 2.849862544 | 0.184903547 | 15.4127 | 1.34E-53 | 1.90E-51 |
| NR1I2 | 1740.178117 | -1.390625575 | 0.26276449 | -5.29229 | 1.21E-07 | 4.24E-07 |
| NR3C2 | 369.6311013 | -1.391537143 | 0.165210431 | -8.42282 | 3.68E-17 | 3.74E-16 |
| NR4A1 | 1600.759504 | -2.131474465 | 0.220938103 | -9.64738 | 5.04E-22 | 8.12E-21 |
| NR4A2 | 526.5174684 | -1.865216233 | 0.233874717 | -7.97528 | 1.52E-15 | 1.31E-14 |
| NR4A3 | 231.197122 | -2.188162166 | 0.239731001 | -9.12757 | 7.01E-20 | 9.23E-19 |
| NR6A1 | 305.1035992 | 1.305595461 | 0.150384003 | 8.681744 | 3.90E-18 | 4.33E-17 |
| NRG1 | 325.0583444 | -1.929450782 | 0.359676208 | -5.36441 | 8.12E-08 | 2.91E-07 |
| OLR1 | 65.92925813 | 1.166636757 | 0.33082095 | 3.52649 | 0.000421 | 0.000916 |
| PDGFA | 1030.401842 | 1.967361873 | 0.18117322 | 10.85901 | 1.81E-27 | 4.60E-26 |
| PDGFRA | 671.0784165 | -1.583607189 | 0.30263038 | -5.23281 | 1.67E-07 | 5.75E-07 |
| PGC | 1353.509785 | 9.350857749 | 0.600329775 | 15.5762 | 1.06E-54 | 1.52E-52 |
| PGF | 339.0869799 | 1.016084209 | 0.162487292 | 6.253315 | 4.02E-10 | 1.90E-09 |
| PGLYRP1 | 2.577503327 | -2.102048653 | 0.220836355 | -9.51858 | 1.76E-21 | 2.69E-20 |
| PI3 | 96.56192526 | 2.435022152 | 0.39689905 | 6.135117 | 8.51E-10 | 3.87E-09 |
| PLXNA1 | 892.1221485 | 1.316404708 | 0.145243394 | 9.063439 | 1.26E-19 | 1.63E-18 |
| PLXNA3 | 643.3854246 | 1.414765824 | 0.169950763 | 8.324563 | 8.46E-17 | 8.33E-16 |
| PLXNC1 | 720.8335511 | 1.847177781 | 0.197120707 | 9.370795 | 7.20E-21 | 1.04E-19 |
| PSMD4 | 11709.84657 | 1.107912906 | 0.111476108 | 9.938568 | 2.83E-23 | 5.05E-22 |
| PTGDR | 17.17657575 | -1.168190029 | 0.196935142 | -5.93185 | 3.00E-09 | 1.27E-08 |
| PTGFR | 274.9866787 | 1.962792923 | 0.353648024 | 5.550131 | 2.85E-08 | 1.08E-07 |
| PTGS2 | 69.29188619 | -2.466026463 | 0.258994695 | -9.52153 | 1.71E-21 | 2.61E-20 |
| PTHLH | 87.53808144 | 2.334310901 | 0.340175415 | 6.86208 | 6.79E-12 | 3.95E-11 |
| RBP4 | 263356.6686 | -1.010639814 | 0.182000831 | -5.55294 | 2.81E-08 | 1.06E-07 |
| REG1A | 1260.311817 | 8.172523103 | 0.654138451 | 12.49357 | 8.09E-36 | 4.02E-34 |
| RFX5 | 1462.059649 | 1.154697384 | 0.102401565 | 11.27617 | 1.72E-29 | 5.36E-28 |
| ROBO1 | 2856.928713 | 2.8891673 | 0.212432684 | 13.60039 | 3.98E-42 | 2.94E-40 |
| S100A12 | 11.56496639 | -3.065086061 | 0.258309668 | -11.8659 | 1.78E-32 | 7.16E-31 |
| S100A6 | 3793.419052 | 1.152290674 | 0.241724386 | 4.766961 | 1.87E-06 | 5.61E-06 |
| S100A8 | 222.0386623 | -1.501489228 | 0.279641228 | -5.36934 | 7.90E-08 | 2.84E-07 |
| S100P | 2483.392115 | 5.74671025 | 0.424825285 | 13.52723 | 1.08E-41 | 7.78E-40 |
| SAA1 | 74934.28716 | -1.960326387 | 0.410649364 | -4.77372 | 1.81E-06 | 5.44E-06 |
| SEMA3F | 913.056021 | 1.278450975 | 0.097177721 | 13.1558 | 1.58E-39 | 1.00E-37 |
| SEMA3G | 457.7035609 | 1.131721414 | 0.169090226 | 6.693003 | 2.19E-11 | 1.20E-10 |
| SEMA4F | 230.4507623 | 1.409895886 | 0.183807123 | 7.670518 | 1.71E-14 | 1.33E-13 |
| SEMA6D | 125.1068851 | -1.156675808 | 0.213723197 | -5.41203 | 6.23E-08 | 2.27E-07 |
| SEMA7A | 435.5899697 | 1.366852172 | 0.207090383 | 6.600269 | 4.10E-11 | 2.18E-10 |
| SERPINA3 | 1106.317179 | -1.090615105 | 0.231376973 | -4.71359 | 2.43E-06 | 7.21E-06 |
| SOCS3 | 2345.90958 | -2.319440771 | 0.221770044 | -10.4588 | 1.34E-25 | 2.88E-24 |
| SORT1 | 2253.000679 | 1.204539391 | 0.156452929 | 7.699053 | 1.37E-14 | 1.07E-13 |
| SPP1 | 28334.76623 | 4.263801948 | 0.366947321 | 11.61966 | 3.27E-31 | 1.18E-29 |
| SRC | 1363.955736 | 1.162706414 | 0.17668431 | 6.5807 | 4.68E-11 | 2.47E-10 |
| STC1 | 597.3173103 | 1.091643223 | 0.195366046 | 5.587681 | 2.30E-08 | 8.82E-08 |
| TEK | 310.8208246 | -1.385506451 | 0.201525028 | -6.87511 | 6.19E-12 | 3.62E-11 |
| TLR4 | 393.9722571 | -1.485362058 | 0.155970587 | -9.52335 | 1.68E-21 | 2.57E-20 |
| TMSB10 | 23744.57358 | 1.455672311 | 0.208342835 | 6.986908 | 2.81E-12 | 1.70E-11 |
| TNFRSF9 | 83.95363346 | 1.423553746 | 0.287028547 | 4.959624 | 7.06E-07 | 2.24E-06 |
| TPM2 | 2044.871668 | 1.956940041 | 0.186604695 | 10.48709 | 9.90E-26 | 2.16E-24 |
| VIPR1 | 324.0662198 | -3.493237319 | 0.248528844 | -14.0557 | 7.11E-45 | 6.19E-43 |

DEGs: differentially expressed genes; TCGA: The Cancer Genome Atlas; LIHC: Liver Hepatocellular Carcinoma; Log2FC: log2FoldChange; lfcSE: standard error of the log2FoldChange estimate; stat = Wald statistic; adj. *p*: adjusted *p* value
