# Supplementary material for: Immune–related biomarkers shared by inflammatory bowel disease and liver cancer
Source: PLoS One. 2022 Apr 22;17(4):e0267358. doi: 10.1371/journal.pone.0267358 (PMC9032416; doi:10.1371/journal.pone.0267358)
Supplement: S2 Table — (DOCX) [file pone.0267358.s006.docx]

**S2 Table. Protein-protein interaction network.**

| Gene | Degree | Betweenness |
| --- | --- | --- |
| IL6 | 61 | 0.212842447 |
| IL10 | 47 | 0.090910269 |
| IL1B | 45 | 0.062539006 |
| CCL2 | 44 | 0.076800099 |
| TLR4 | 39 | 0.038482736 |
| MMP9 | 35 | 0.03179018 |
| HGF | 33 | 0.138254703 |
| CXCL12 | 31 | 0.050396688 |
| PTGS2 | 29 | 0.035707909 |
| CSF3 | 29 | 0.011924017 |
| SRC | 28 | 0.046820238 |
| FOS | 26 | 0.060825403 |
| SPP1 | 25 | 0.01548725 |
| CCL4 | 25 | 0.013496163 |
| CXCL2 | 24 | 0.006991788 |
| CSF1R | 24 | 0.014543556 |
| SAA1 | 23 | 0.037864049 |
| CCL20 | 23 | 0.004243448 |
| LCN2 | 22 | 0.041209254 |
| CCR1 | 22 | 0.025737763 |
| IL1RN | 21 | 0.00530061 |
| IL11 | 21 | 0.008015347 |
| HMOX1 | 21 | 0.007372942 |
| PI3 | 19 | 0.002842243 |
| SOCS3 | 18 | 0.002574405 |
| PGF | 17 | 0.005688233 |
| S100A8 | 15 | 0.0466968 |
| IL2RB | 15 | 0.006496106 |
| FPR1 | 15 | 0.016999365 |
| CAT | 15 | 0.02063104 |
| C5AR1 | 15 | 0.020007004 |
| FCGR2B | 14 | 8.71E-04 |
| S100A12 | 13 | 0.002264023 |
| PTHLH | 13 | 0.023208243 |
| IL33 | 13 | 9.36E-04 |
| TEK | 12 | 4.30E-04 |
| CD4 | 12 | 0.015874159 |
| CD14 | 12 | 8.67E-04 |
| ADM | 12 | 0.025231116 |
| PDGFA | 11 | 0.003907411 |
| NOX4 | 11 | 5.23E-04 |
| PDGFRA | 10 | 3.51E-04 |
| GCGR | 10 | 0.007392008 |
| CD1D | 10 | 2.46E-04 |
| ROBO1 | 8 | 0.008317415 |
| PTGFR | 8 | 0.005417632 |
| PLXNA1 | 8 | 0.004677544 |
| IL6ST | 8 | 0.011376432 |
| GLP1R | 8 | 0.022755137 |
| EDNRB | 8 | 0.002846044 |
| PLXNC1 | 7 | 0.004645188 |
| PLXNA3 | 7 | 0.00450272 |
| OLR1 | 7 | 0.008333392 |
| MMP12 | 7 | 1.21E-05 |
| IL1RL1 | 7 | 2.59E-04 |
| IL1RAP | 7 | 1.17E-04 |
| IL18R1 | 7 | 2.59E-04 |
| GHR | 7 | 2.04E-04 |
| DUOX2 | 7 | 1.51E-04 |
| CCL23 | 7 | 0 |
| BMP7 | 7 | 0.001032163 |
| SEMA3F | 6 | 0.001285513 |
| RBP4 | 6 | 0.027622965 |
| NR4A1 | 6 | 0.003771089 |
| EDN3 | 6 | 6.41E-04 |
| DUOX1 | 6 | 0 |
| DKK1 | 6 | 0.019722299 |
| AVPR1A | 6 | 6.41E-04 |
| VIPR1 | 5 | 0 |
| TNFRSF9 | 5 | 4.57E-05 |
| SEMA7A | 5 | 2.35E-04 |
| SEMA6D | 5 | 2.35E-04 |
| SEMA3G | 5 | 2.35E-04 |
| PTGDR | 5 | 0 |
| NR4A2 | 5 | 0.016452327 |
| IL13RA2 | 5 | 1.00E-04 |
| SEMA4F | 4 | 9.24E-05 |
| PGLYRP1 | 4 | 0 |
| NRG1 | 4 | 8.56E-04 |
| LCN1 | 4 | 0.002244368 |
| KIR2DL1 | 4 | 9.40E-04 |
| NR4A3 | 3 | 0 |
| AZGP1 | 3 | 4.55E-04 |
| S100A6 | 2 | 0.019607843 |
| NR6A1 | 2 | 4.66E-04 |
| NR1I2 | 2 | 0 |
| NDRG1 | 2 | 0.003847082 |
| MICB | 2 | 2.34E-04 |
| GIG25 | 2 | 0 |
| GBP2 | 2 | 0 |
| CMTM6 | 2 | 1.16E-04 |
| CD81 | 2 | 2.73E-04 |
| TMSB10 | 1 | 0 |
| STC1 | 1 | 0 |
| SORT1 | 1 | 0 |
| REG1A | 1 | 0 |
| PSMD4 | 1 | 0 |
| PGC | 1 | 0 |
| NR3C2 | 1 | 0 |
| MASP1 | 1 | 0 |
| LGR5 | 1 | 0 |
| INHBA | 1 | 0 |
| CRABP1 | 1 | 0 |
| BMP8B | 1 | 0 |
| BMP8A | 1 | 0 |
| BLNK | 1 | 0 |
| ARG2 | 1 | 0 |
| TPM2 | 0 | 0 |
| S100P | 0 | 0 |
| RFX5 | 0 | 0 |
| KLKB1 | 0 | 0 |
| BIRC5 | 0 | 0 |
