# Supplementary material for: Immune–related biomarkers shared by inflammatory bowel disease and liver cancer
Source: PLoS One. 2022 Apr 22;17(4):e0267358. doi: 10.1371/journal.pone.0267358 (PMC9032416; doi:10.1371/journal.pone.0267358)
Supplement: S3 Table — (DOCX) [file pone.0267358.s007.docx]

**S3 Table. The association of the top 20 hub genes and overall survival in the TCGA_LIHC dataset.**

| **Symbol** | **Univariate Cox regression analysis** | | |
| --- | --- | --- | --- |
|  | **HR** | **95% CI** | ***p*-value** |
| IL6 | 0.95 | 0.671-1.338 | >0.05 |
| IL10 | 1.05 | 0.742-1.479 | >0.05 |
| IL1B | 1.32 | 0.932-1.879 | >0.05 |
| CCL2 | 0.75 | 0.532-1.066 | >0.05 |
| TLR4 | 0.85 | 0.604-1.207 | >0.05 |
| MMP9 | 1.48 | 1.041-2.091 | 0.028 |
| HGF | 0.79 | 0.559-1.119 | >0.05 |
| CXCL12 | 0.88 | 0.62-1.236 | >0.05 |
| PTGS2 | 1.00 | 0.709-1.415 | >0.05 |
| CSF3 | 0.75 | 0.532-1.065 | >0.05 |
| SRC | 1.46 | 1.033-2.064 | 0.032 |
| FOS | 1.13 | 0.799-1.596 | >0.05 |
| SPP1 | 1.99 | 1.396-2.849 | 0.0001 |
| CCL4 | 1.10 | 0.779-1.558 | >0.05 |
| CXCL2 | 0.67 | 0.476-0.955 | 0.026 |
| CSF1R | 0.95 | 0.675-1.348 | >0.05 |
| SAA1 | 0.93 | 0.655-1.308 | >0.05 |
| CCL20 | 1.23 | 0.867-1.733 | >0.05 |
| LCN2 | 0.85 | 0.602-1.201 | >0.05 |
| CCR1 | 1.03 | 0.729-1.455 | >0.05 |

HR: Hazard ratio; CI: confident interval; TCGA: The Cancer Genome Atlas; LIHC: Liver Hepatocellular Carcinoma
