# Supplementary material for: Immune–related biomarkers shared by inflammatory bowel disease and liver cancer
Source: PLoS One. 2022 Apr 22;17(4):e0267358. doi: 10.1371/journal.pone.0267358 (PMC9032416; doi:10.1371/journal.pone.0267358)
Supplement: S4 Table — (DOCX) [file pone.0267358.s008.docx]

**S4 Table. Gene-transcription factor interaction network.**

| Label | Degree | Betweenness |
| --- | --- | --- |
| CXCL2 | 11 | 215.69 |
| SPP1 | 10 | 184.98 |
| MMP9 | 7 | 124.36 |
| SRC | 7 | 112.98 |
| FOXC1 | 4 | 173.81 |
| FOXL1 | 2 | 38.64 |
| GATA2 | 2 | 24.39 |
| YY1 | 2 | 22.31 |
| ZNF354C | 2 | 19.79 |
| TP53 | 2 | 14.06 |
| PDX1 | 1 | 0 |
| CEBPB | 1 | 0 |
| FOS | 1 | 0 |
| JUN | 1 | 0 |
| MEF2A | 1 | 0 |
| POU2F2 | 1 | 0 |
| HOXA5 | 1 | 0 |
| NFIC | 1 | 0 |
| SRF | 1 | 0 |
| HNF4A | 1 | 0 |
| HINFP | 1 | 0 |
| E2F6 | 1 | 0 |
| NFKB1 | 1 | 0 |
| MAX | 1 | 0 |
| USF1 | 1 | 0 |
| USF2 | 1 | 0 |
| SREBF1 | 1 | 0 |
| STAT1 | 1 | 0 |
| RUNX2 | 1 | 0 |
| NR3C1 | 1 | 0 |
| HNF1B | 1 | 0 |
