# Supplementary material for: Immune–related biomarkers shared by inflammatory bowel disease and liver cancer
Source: PLoS One. 2022 Apr 22;17(4):e0267358. doi: 10.1371/journal.pone.0267358 (PMC9032416; doi:10.1371/journal.pone.0267358)
Supplement: S5 Table — (DOCX) [file pone.0267358.s009.docx]

**S5 Table. Gene-microRNA interaction network.**

| Label | Degree | Betweenness |
| --- | --- | --- |
| CXCL2 | 44 | 2486.13 |
| MMP9 | 28 | 1412.48 |
| SRC | 27 | 1835.63 |
| SPP1 | 22 | 919.75 |
| hsa-mir-124-3p | 4 | 693.41 |
| hsa-mir-34a-5p | 3 | 366.42 |
| hsa-mir-1-3p | 3 | 366.42 |
| hsa-mir-7-5p | 3 | 104.67 |
| hsa-mir-99b-5p | 3 | 104.67 |
| hsa-mir-155-5p | 2 | 206.1 |
| hsa-mir-146a-5p | 2 | 116.53 |
| hsa-mir-128-3p | 2 | 43.79 |
| hsa-mir-138-5p | 2 | 43.79 |
| hsa-mir-141-3p | 2 | 43.79 |
| hsa-mir-200a-3p | 2 | 43.79 |
| hsa-mir-101-3p | 2 | 43.79 |
| hsa-mir-214-3p | 2 | 43.79 |
| hsa-mir-335-5p | 2 | 33.5 |
| hsa-mir-126-3p | 2 | 33.5 |
| hsa-mir-129-2-3p | 2 | 33.5 |
| hsa-mir-147a | 2 | 33.5 |
| hsa-mir-16-5p | 2 | 33.5 |
| hsa-mir-27a-3p | 2 | 33.5 |
| hsa-mir-27a-5p | 2 | 33.5 |
| hsa-mir-145-5p | 2 | 27.38 |
| hsa-mir-195-5p | 2 | 27.38 |
| hsa-mir-330-3p | 2 | 27.38 |
| hsa-mir-374a-5p | 2 | 27.38 |
| hsa-mir-21-5p | 1 | 0 |
| hsa-mir-31-5p | 1 | 0 |
| hsa-mir-98-5p | 1 | 0 |
| hsa-mir-205-5p | 1 | 0 |
| hsa-mir-9-5p | 1 | 0 |
| hsa-mir-33b-5p | 1 | 0 |
| hsa-mir-151a-5p | 1 | 0 |
| hsa-mir-15b-3p | 1 | 0 |
| hsa-mir-181b-5p | 1 | 0 |
| hsa-mir-181d-5p | 1 | 0 |
| hsa-mir-192-5p | 1 | 0 |
| hsa-mir-215-5p | 1 | 0 |
| hsa-mir-217 | 1 | 0 |
| hsa-mir-27b-3p | 1 | 0 |
| hsa-mir-302d-5p | 1 | 0 |
| hsa-mir-323a-3p | 1 | 0 |
| hsa-mir-34a-3p | 1 | 0 |
| hsa-mir-371a-5p | 1 | 0 |
| hsa-mir-449a | 1 | 0 |
| hsa-mir-603 | 1 | 0 |
| hsa-mir-765 | 1 | 0 |
| hsa-mir-769-5p | 1 | 0 |
| hsa-mir-20a-5p | 1 | 0 |
| hsa-mir-21-3p | 1 | 0 |
| hsa-mir-210-3p | 1 | 0 |
| hsa-mir-671-5p | 1 | 0 |
| hsa-mir-373-3p | 1 | 0 |
| hsa-mir-372-3p | 1 | 0 |
| hsa-mir-148b-3p | 1 | 0 |
| hsa-mir-133a-3p | 1 | 0 |
| hsa-mir-103a-3p | 1 | 0 |
| hsa-mir-107 | 1 | 0 |
| hsa-mir-218-5p | 1 | 0 |
| hsa-mir-223-3p | 1 | 0 |
| hsa-mir-23b-3p | 1 | 0 |
| hsa-mir-376c-3p | 1 | 0 |
| hsa-mir-377-3p | 1 | 0 |
| hsa-mir-494-3p | 1 | 0 |
| hsa-mir-497-5p | 1 | 0 |
| hsa-mir-655-3p | 1 | 0 |
| hsa-mir-126-5p | 1 | 0 |
| hsa-mir-181a-5p | 1 | 0 |
| hsa-mir-361-5p | 1 | 0 |
| hsa-mir-484 | 1 | 0 |
| hsa-mir-495-3p | 1 | 0 |
| hsa-mir-10b-5p | 1 | 0 |
| hsa-mir-182-5p | 1 | 0 |
| hsa-mir-941 | 1 | 0 |
| hsa-let-7a-5p | 1 | 0 |
| hsa-mir-17-3p | 1 | 0 |
| hsa-mir-188-5p | 1 | 0 |
| hsa-mir-193b-3p | 1 | 0 |
| hsa-mir-194-3p | 1 | 0 |
| hsa-mir-221-5p | 1 | 0 |
| hsa-mir-22-5p | 1 | 0 |
| hsa-mir-2355-5p | 1 | 0 |
| hsa-mir-3065-3p | 1 | 0 |
| hsa-mir-3127-3p | 1 | 0 |
| hsa-mir-3157-5p | 1 | 0 |
| hsa-mir-32-5p | 1 | 0 |
| hsa-mir-33a-3p | 1 | 0 |
| hsa-mir-423-5p | 1 | 0 |
| hsa-mir-4525 | 1 | 0 |
| hsa-mir-491-5p | 1 | 0 |
| hsa-mir-5008-5p | 1 | 0 |
| hsa-mir-92a-3p | 1 | 0 |
| hsa-mir-92b-3p | 1 | 0 |
