# Supplementary material for: Immune–related biomarkers shared by inflammatory bowel disease and liver cancer
Source: PLoS One. 2022 Apr 22;17(4):e0267358. doi: 10.1371/journal.pone.0267358 (PMC9032416; doi:10.1371/journal.pone.0267358)
Supplement: S6 Table — (DOCX) [file pone.0267358.s010.docx]

**S6 Table. Gene-drug interaction network of SRC.**

| Label | Degree | Betweenness |
| --- | --- | --- |
| SRC | 46 | 1035 |
| Dasatinib | 1 | 0 |
| RU84687 | 1 | 0 |
| RU79256 | 1 | 0 |
| N6-Benzyl Adenosine-5'-Diphosphate | 1 | 0 |
| RU85493 | 1 | 0 |
| RU78262 | 1 | 0 |
| Phosphonotyrosine | 1 | 0 |
| Malonic acid | 1 | 0 |
| RU83876 | 1 | 0 |
| RU90395 | 1 | 0 |
| RU79072 | 1 | 0 |
| RU78783 | 1 | 0 |
| 1-Tert-Butyl-3-(4-Chloro-Phenyl)-1h-Pyrazolo[3,4-D]Pyrimidin-4-Ylamine | 1 | 0 |
| PASBN | 1 | 0 |
| RU82129 | 1 | 0 |
| PAS219 | 1 | 0 |
| DPI59 | 1 | 0 |
| RU82197 | 1 | 0 |
| Phenylphosphate | 1 | 0 |
| RU78300 | 1 | 0 |
| RU79073 | 1 | 0 |
| RU82209 | 1 | 0 |
| ISO24 | 1 | 0 |
| RU85053 | 1 | 0 |
| RU78299 | 1 | 0 |
| Oxalic Acid | 1 | 0 |
| RU78191 | 1 | 0 |
| Citric Acid | 1 | 0 |
| RU81843 | 1 | 0 |
| 4-[(4-Methyl-1-piperazinyl)methyl]-N-[3-[[4-(3-pyridinyl)-2-pyrimidinyl]amino]phenyl]-benzamide | 1 | 0 |
| Purvalanol A | 1 | 0 |
| XL228 | 1 | 0 |
| Bosutinib | 1 | 0 |
| 1-[1-(3-Aminophenyl)-3-Tert-Butyl-1h-Pyrazol-5-Yl]-3-Naphthalen-1-Ylurea | 1 | 0 |
| 1-[1-(3-Aminophenyl)-3-Tert-Butyl-1h-Pyrazol-5-Yl]-3-Phenylurea | 1 | 0 |
| 3-[4-Amino-1-(1-Methylethyl)-1h-Pyrazolo[3,4-D]pyrimidin-3-Yl]phenol | 1 | 0 |
| N-[4-(3-Bromo-phenylamino)-quinazolin-6-yl]-acrylamide | 1 | 0 |
| [4-({4-[(5-cyclopropyl-1H-pyrazol-3-yl)amino]quinazolin-2-yl}amino)phenyl]acetonitrile | 1 | 0 |
| 1-cyclopentyl-3-(1H-pyrrolo[2,3-b]pyridin-5-yl)-1H-pyrazolo[3,4-d]pyrimidin-4-amine | 1 | 0 |
| 1-cyclobutyl-3-(3,4-dimethoxyphenyl)-1H-pyrazolo[3,4-d]pyrimidin-4-amine | 1 | 0 |
| 1-(1-Methylethyl)-3-Quinolin-6-Yl-1h-Pyrazolo[3,4-D]pyrimidin-4-Amine | 1 | 0 |
| 2-(4-Carcoxy-5-Isopropylthiazolyl)Benzopiperidine | 1 | 0 |
| N-(4-Phenylamino-Quinazolin-6-Yl)-Acrylamide | 1 | 0 |
| (2E)-N-{4-[(3-Bromophenyl)amino]quinazolin-6-Yl}-4-(Dimethylamino)but-2-Enamide | 1 | 0 |
| Ponatinib | 1 | 0 |
| Nintedanib | 1 | 0 |
