# Supplementary material for: Immune–related biomarkers shared by inflammatory bowel disease and liver cancer
Source: PLoS One. 2022 Apr 22;17(4):e0267358. doi: 10.1371/journal.pone.0267358 (PMC9032416; doi:10.1371/journal.pone.0267358)
Supplement: S7 Table — (DOCX) [file pone.0267358.s011.docx]

**S7 Table. Gene-drug interaction network of MMP9.**

| Label | Degree | Betweenness |
| --- | --- | --- |
| MMP9 | 11 | 55 |
| Marimastat | 1 | 0 |
| Minocycline | 1 | 0 |
| Captopril | 1 | 0 |
| Glucosamine | 1 | 0 |
| 2-Amino-N,3,3-Trimethylbutanamide | 1 | 0 |
| 2-{[Formyl(hydroxy)amino]methyl}-4-methylpentanoic acid | 1 | 0 |
| AE-941 | 1 | 0 |
| PG-530742 | 1 | 0 |
| 5-(4-Phenoxyphenyl)-5-(4-Pyrimidin-2-Ylpiperazin-1-Yl)pyrimidine-2,4,6(2h,3h)-Trione | 1 | 0 |
| (2r)-2-Amino-3,3,3-Trifluoro-N-Hydroxy-2-{[(4-Phenoxyphenyl)sulfonyl]methyl}propanamide | 1 | 0 |
| (3r)-4,4-Difluoro-3-[(4-Methoxyphenyl)sulfonyl]butanoic Acid | 1 | 0 |
