# Supplementary material for: Immune–related biomarkers shared by inflammatory bowel disease and liver cancer
Source: PLoS One. 2022 Apr 22;17(4):e0267358. doi: 10.1371/journal.pone.0267358 (PMC9032416; doi:10.1371/journal.pone.0267358)
Supplement: S8 Table — (DOCX) [file pone.0267358.s012.docx]

**S8 Table. Gene-disease interaction network.**

| Label | Degree | Betweenness |
| --- | --- | --- |
| SPP1 | 34 | 1534.5 |
| CXCL2 | 19 | 919.5 |
| SRC | 14 | 754 |
| Mammary Neoplasms | 3 | 895.33 |
| Pulmonary Fibrosis | 2 | 181.33 |
| Dermatitis, Allergic Contact | 2 | 181.33 |
| Rheumatoid Arthritis | 1 | 0 |
| Bone Resorption | 1 | 0 |
| Cholestasis | 1 | 0 |
| Esophageal Neoplasms | 1 | 0 |
| Heart failure | 1 | 0 |
| Hypertensive disease | 1 | 0 |
| Inflammation | 1 | 0 |
| Chronic Obstructive Airway Disease | 1 | 0 |
| Degenerative polyarthritis | 1 | 0 |
| Reperfusion Injury | 1 | 0 |
| Shock, Hemorrhagic | 1 | 0 |
| Dermatologic disorders | 1 | 0 |
| Myocardial Ischemia | 1 | 0 |
| Acute Lung Injury | 1 | 0 |
| Arsenic Poisoning | 1 | 0 |
| Infarction, Middle Cerebral Artery | 1 | 0 |
| Adenocarcinoma | 1 | 0 |
| Asbestosis | 1 | 0 |
| Brain Neoplasms | 1 | 0 |
| Calcinosis | 1 | 0 |
| Glioma | 1 | 0 |
| Focal glomerulosclerosis | 1 | 0 |
| Heart Diseases | 1 | 0 |
| Heart valve disease | 1 | 0 |
| Hypersensitivity | 1 | 0 |
| Kidney Calculi | 1 | 0 |
| Kidney Diseases | 1 | 0 |
| Liver Cirrhosis | 1 | 0 |
| Alcoholic Liver Diseases | 1 | 0 |
| Liver Neoplasms, Experimental | 1 | 0 |
| Lung Neoplasms | 1 | 0 |
| Mammary Neoplasms, Experimental | 1 | 0 |
| Mesothelioma | 1 | 0 |
| Neoplasm Metastasis | 1 | 0 |
| Neoplasms, Experimental | 1 | 0 |
| Pancreatic Diseases | 1 | 0 |
| Pleural Neoplasms | 1 | 0 |
| Pneumonia | 1 | 0 |
| Proteinuria | 1 | 0 |
| Adverse reaction to drug | 1 | 0 |
| Uremia | 1 | 0 |
| Lytic lesion | 1 | 0 |
| Leukoencephalopathies | 1 | 0 |
| Diabetic Cardiomyopathies | 1 | 0 |
| Acute kidney injury | 1 | 0 |
| Cerebral Hemorrhage | 1 | 0 |
| Chemical and Drug Induced Liver Injury | 1 | 0 |
| Bladder Neoplasm | 1 | 0 |
| Renal Cell Carcinoma | 1 | 0 |
| Colitis | 1 | 0 |
| Colonic Neoplasms | 1 | 0 |
| Cardiomegaly | 1 | 0 |
| Neoplasm Invasiveness | 1 | 0 |
| Status Epilepticus | 1 | 0 |
| Stomach Neoplasms | 1 | 0 |
| Autosomal Recessive Polycystic Kidney Disease | 1 | 0 |
| Transitional cell carcinoma of bladder | 1 | 0 |
| Leiomyosarcoma of uterus | 1 | 0 |
| Increased gastric cancer | 1 | 0 |
| Hereditary nonpolyposis colorectal carcinoma | 1 | 0 |
